# Jerantinine A induces tumor-specific cell death through modulation of splicing factor 3b subunit 1 (SF3B1)

**DOI:** 10.1038/srep42504

**Published:** 2017-02-15

**Authors:** Felicia Fei-Lei Chung, Perry Faith Tze Ming Tan, Vijay Joseph Raja, Boon-Shing Tan, Kuan-Hon Lim, Toh-Seok Kam, Ling-Wei Hii, Si Hoey Tan, Sze-Jia See, Yuen-Fen Tan, Li-Zhe Wong, Wai Keat Yam, Chun Wai Mai, Tracey D. Bradshaw, Chee-Onn Leong

**Affiliations:** 1 Center for Cancer and Stem Cell Research, International Medical University, Bukit Jalil, 57000 Kuala Lumpur, Malaysia; 2 School of Medicine, International Medical University, Bukit Jalil, 57000 Kuala Lumpur, Malaysia; 3 Department of Biochemistry, Weill Cornell Medical College, New York, NY 10021, USA; 4 Institute of Biological Chemistry, Academia Sinica, Taipei, Taiwan; 5 School of Pharmacy, University of Nottingham Malaysia Campus, Jalan Broga, 43500 Semenyih, Selangor, Malaysia; 6 Department of Chemistry, Faculty of Science, University of Malaya, 50603 Kuala Lumpur, Malaysia; 7 School of Postgraduate Studies, International Medical University, Bukit Jalil, 57000 Kuala Lumpur, Malaysia; 8 School of Pharmacy, International Medical University, Bukit Jalil, 57000 Kuala Lumpur, Malaysia; 9 School of Pharmacy, University of Nottingham, University Park, Nottingham NG7 2RD, UK

## Abstract

Precursor mRNA (pre-mRNA) splicing is catalyzed by a large ribonucleoprotein complex known as the spliceosome. Numerous studies have indicated that aberrant splicing patterns or mutations in spliceosome components, including the splicing factor 3b subunit 1 (SF3B1), are associated with hallmark cancer phenotypes. This has led to the identification and development of small molecules with spliceosome-modulating activity as potential anticancer agents. Jerantinine A (JA) is a novel indole alkaloid which displays potent anti-proliferative activities against human cancer cell lines by inhibiting tubulin polymerization and inducing G2/M cell cycle arrest. Using a combined pooled-genome wide shRNA library screen and global proteomic profiling, we showed that JA targets the spliceosome by up-regulating SF3B1 and SF3B3 protein in breast cancer cells. Notably, JA induced significant tumor-specific cell death and a significant increase in unspliced pre-mRNAs. In contrast, depletion of endogenous SF3B1 abrogated the apoptotic effects, but not the G2/M cell cycle arrest induced by JA. Further analyses showed that JA stabilizes endogenous SF3B1 protein in breast cancer cells and induced dissociation of the protein from the nucleosome complex. Together, these results demonstrate that JA exerts its antitumor activity by targeting SF3B1 and SF3B3 in addition to its reported targeting of tubulin polymerization.

Precursor mRNA (pre-mRNA) splicing is a fundamental process in eukaryotic cells, which is catalyzed by the spliceosome, a macromolecular ribonucleoprotein (RNP) complex composed of five small nuclear ribonucleoproteins (U1, U2, U4, U5 and U6 snRNPs) and more than 200 polypeptides^1^,^2^,^3^. The splicing factor 3b subunit 1 (SF3B1) protein is a core component of the U2 snRNP at the catalytic center of the spliceosome, which recognizes and defines the 3′ splice site at the intron-exon junctions^4^.

Through pre-mRNA splicing, a single pre-mRNA transcript may give rise to multiple different combinations of introns and exons, resulting in increased transcript diversity and the synthesis of alternative proteins^5^. While changes in alternative splicing patterns play an integral role in normal development and cell differentiation, numerous cancer-specific aberrant splicing patterns have been documented^6^,^7^. However, it is currently unclear whether the observed splicing abnormalities are a by-product of cellular transformation or an intrinsic characteristic of transformed cells.

Recently, growing evidence has demonstrated that aberrant splicing contributes to essential phenotypes associated with transformed cells. For instance, alternative protein products of epidermal growth factor receptor (EGFR)^8^, p53^9^, vascular endothelial growth factor (VEGF)^10^, and E-cadherin^11^ reportedly promoted cancer-associated pathways, including the evasion of apoptosis, increased cell proliferation, angiogenesis, and invasion. Mutations in SF3B1 have also been reported in myelodysplastic syndromes (MDS) as well as numerous cancers, including acute myeloid leukemia, primary myelofibrosis, chronic myelomonocytic leukemia (CML)^12^, chronic lymphocytic leukemia (CLL)^13^,^14^, multiple myeloma, uveal melanoma^15^,^16^,^17^,^18^ and breast cancers^19^,^20^,^21^. While it is currently unclear as to how SF3B1 mutations might alter its function, previous studies have shown that the dysregulation of spliceosomal components can alter splicing patterns, causing intron retention or exon skipping, and affect protein isoform balances leading to abnormal cell proliferation or differentiation^2^,^22^. As such, the spliceosome has emerged as an attractive target for anticancer treatment. Several spliceosome modulators have already been identified, including natural products derived from bacterial fermentation (e.g. pladienolides, GEX1, FR901463, etc.) and their synthetic analogues (spliceostatin A, meayamycin and E7107) as well as natural plant products (e.g. isoginkgetin)^23^.

Indole alkaloids represent a large and highly structurally diverse group of secondary metabolites with remarkable bioactivities against the different targets in cancer. The importance of this group of compounds is best represented by the Vinca alkaloid vinblastine, which is currently among the foremost drugs used in cancer chemotherapy^24^. Previously, we have described the potent and selective antitumor activity of seven new *Aspidosperma* indole alkaloids, jerantinines A-G, isolated from the leaf extracts of the Malayan plant *Tabernaemontana corymbosa* (Fig. 1A)^25^. Jerantinines A-E were found to display pronounced *in vitro* anti-proliferative activities against human cancer cell lines in the nanomolar range^26^,^27^,^28^. Furthermore, we have recently demonstrated that jerantinine A and B and the acetate derivative inhibited tubulin polymerization, polo-like kinase 1 (PLK1) activity and induced G2/M cell cycle arrest in a panel of human cancer cell lines consisting of vincristine-resistant nasopharyngeal carcinoma cells^25^, as well as breast, colorectal, lung and pancreatic carcinoma cells^27^,^28^. Similarly, jerantinine E was also shown to disrupt microtubules, and displayed significant antitumor activity against human cervical carcinoma cells^29^. Importantly, no cross-resistance to jerantinines was observed in vincristine-resistant HCT-116 cells, suggesting that jerantinines overcome p-glycoprotein-mediated multidrug resistance and might affect other cancer-relevant targets besides tubulin^25^,^27^,^28^.

Using a pooled-genome wide shRNA library screen and global proteomic profiling, we demonstrated that jerantinine A (JA) targets the cancer spliceosome through the upregulation of SF3B1 and SF3B3 proteins in breast cancer cells. Importantly, ectopic expression of SF3B1, SF3B3 or JA treatment induced significant tumor-specific cell death accompanied by the accumulation of unspliced pre-mRNAs. In contrast, the depletion of endogenous SF3B1 or SF3B3 abrogated the apoptotic effects induced by JA, but not the G2/M cell cycle arrest. Further analyses revealed that JA stabilizes endogenous SF3B1 protein and disrupts the binding of the protein to the nucleosome complex in breast cancer cells. Together, our results suggest that JA exerts its antitumor activity by targeting SF3B1 in addition to its reported targeting of tubulin polymerization.

## Results

### Jerantinine A induces tumor-specific cell death in breast cancer cell lines

**Figure 1:**
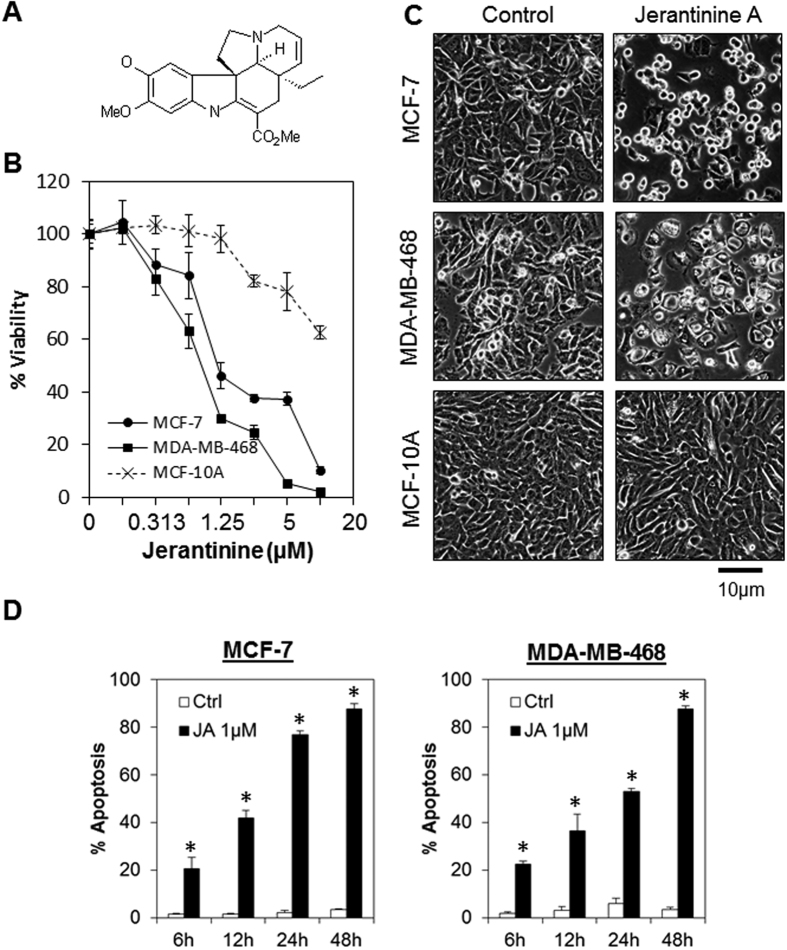
JA induces tumor-specific cell death in breast cancer cell lines. (**A**) Chemical structure of JA. (**B**) Growth inhibitory effects of JA on breast cancer cells. MCF-7, and MDA-MB-468 breast cancer cell lines, as well as the non-transformed MCF-10A breast cell line, were treated with increasing concentrations of JA. Cell viability was determined using the MTT cell viability assay 72 h after JA treatment. Each data point represents the mean ± s.d. of at least 3 independent experiments. (**C**) Morphological changes at 24 h following JA treatment in MCF-7, MDA-MB-468, and MCF-10A cells. Original magnification, x100. (**D**) JA induced time-dependent apoptosis in MCF-7 and MDA-MB-468 cells. Cells were treated with 1 μM of JA followed by quantitation of apoptosis at various time points using annexin V/7-AAD flow cytometry. Bars represent the means ± s.d. of 3 independent experiments. Asterisks (*) indicate statistical significance compared with vehicle cells (P < 0.01, Student’s *t*-test).

To test the selective antitumor activity of JA, we compared its anti-proliferative activities in a panel of breast cancer cell lines consisting of estrogen receptor (ER)-positive (MCF-7 and T47D) and triple-negative cells (MDA-MB-468). As shown in Fig. 1B and Supplementary Table S1, JA elicits activity against all the breast cancer cell lines being tested (approx. IC_50_ = 1 μM), while the non-transformed MCF-10A cells were relatively resistant (IC_50_ > 10 μM). Similarly, apoptotic morphological changes were also observed in JA-treated cancer cells, but no such changes were observed in non-transformed cells (Fig. 1C). The effects of JA on cell cycle arrest and cell death were further investigated by propidium iodide staining and annexin V/7-AAD flow cytometry analysis, respectively. Consistent with our previous reports, JA induced significant G2/M arrest in MCF-7 cells (19% in control cells vs 64% in JA-treated cells, Supplementary Fig. S1A)^27^,^28^. The percentage of apoptotic cells in MCF-7 and MDA-MB-468 cells after JA treatment was also significantly higher than in the control cells (P < 0.01, Student’s t-test; Fig. 1D). However, no significant induction of caspase 3/7, 8 and 9 activities were detected in response to JA treatment, suggesting that JA might induce caspase-independent apoptosis (Supplementary Fig. S1B). Of note, the induction of apoptosis was observed as early as 6 h after treatment, suggesting that JA might target endogenous proteins rather than transcriptional activation.

### Proteomic profiling of MCF-7 breast cancer cells following JA treatment

We have previously demonstrated that jerantinine A and B inhibited tubulin polymerization, PLK1 activity and induced G2/M cell cycle arrest in a panel of human cancer cell lines^27^,^28^. Importantly, no cross-resistance to jerantinine A and B was observed in vincristine-resistant cancer cells, suggesting that jerantinine A and B might affect other cancer-relevant targets in addition to tubulin. To systematically identify molecular targets involved in modulating the antitumor activity of JA, we carried out a combined proteomic-RNAi screen. We hypothesized that direct targets mediating the antitumor activities of JA should have two properties: 1) they should be differentially expressed following JA treatment, and 2) they should be functionally required to sustain the antitumor activity of JA.

**Figure 2:**
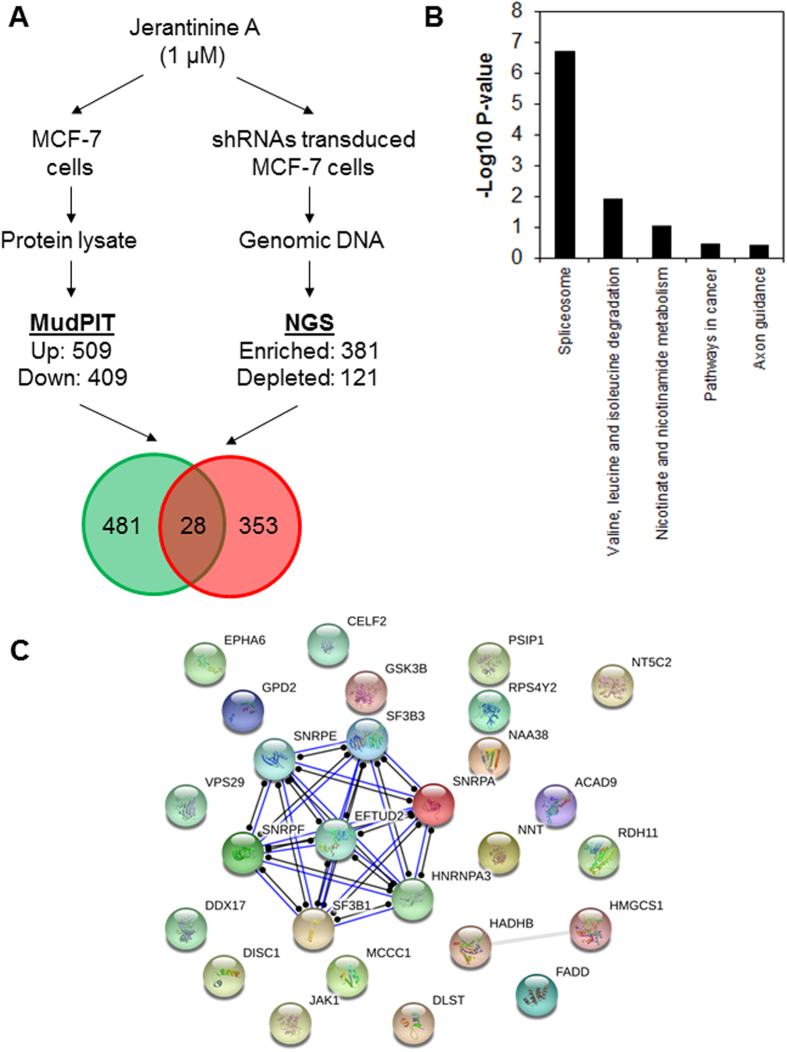
Combined proteomic-RNAi screen analyses revealed spliceosome as putative target of JA. (**A**) Design of the combined proteomic profiling and genome-wide shRNA screen to identify new molecular targets of JA. MCF-7 cells were treated with 1 μM of JA for 8 h and their protein lysate were isolated for global proteomic analysis by MudPIT (left). Genes that were required for JA activities were identified by pooled genome-wide shRNA screen and NGS analysis following treatment of MCF-7 cells with 1 μM of JA for 72 h (right). The intersection of the data generated 28 candidate targets that were required for JA activities and were up-regulated in JA-treated cells. (**B**) DAVID enrichment analysis showing JA target genes associated with the spliceosome pathway (KEGG pathway, P < 0.01). (**C**) Protein-protein interaction network of the JA target proteins. Networks were generated with STRING at the highest confidence threshold (0.9). Note the seven interacting targets (SF3B1, SF3B3, SNRPA, SNRPE, SNRPF, HNRNPA3 and EFTUD2) are proteins within the spliceosome complex.

To identify proteins that are differentially regulated in JA-treated MCF-7 cells, we conducted a global proteomic analysis using multidimensional protein identification technology (MudPIT) (Fig. 2A and Supplementary Fig. S2A). We identified 509 up-regulated proteins (Supplementary Table S2) and 429 down-regulated proteins (Supplementary Table S3). Among them were proteins that are involved in assembly of the spliceosome, cellular metabolism, endocytosis and axon guidance (Supplementary Fig. S2B). Of note, several proteins that are associated with mitosis (e.g. ANAPC7, MCM3, MCM7, SMC2, SMC3, TUBG1 and TUBG2) and apoptosis (e.g. FADD and DIABLO) were also found to be up-regulated, consistent with the phenotype we observed previously^27^,^28^.

### Genome-wide shRNA functional screen identifies a compendium of genes affecting sensitivity to JA

In a parallel RNAi screen, we used the same cell line used for the proteomic profiling to identify genes that were functionally required for JA activity. We treated the pooled genome-wide shRNA transduced cells for 72 h with JA, isolated the genomic DNA and analyzed the abundance of shRNA by next-generation sequencing (NGS). Conceptually, the shRNAs that are “enriched” (Z-scores >2) in the surviving cell population are genes that are essential for eliciting the killing response caused by JA since silencing the gene prevents the cell-death signal from propagating. Thus, analysis of the “enriched” shRNAs indicates which genes are necessary for the lethal activity of JA. Vice versa, shRNAs that make cells more susceptible to JA treatment would be “depleted” in the surviving cell population, indicating that these genes might help cells survive in the presence of JA. Hence, inhibiting the function of these genes might sensitize the antitumor effects of JA.

Following this screen, a total of 381 candidate genes that were enriched in JA-treated cells and 121 candidate genes that were depleted in JA-treated cells were identified (Fig. 2A and Supplementary Fig. S3A,B). Among the shRNAs that were highly enriched in JA-treated cells are genes that are involved in the human spliceosome, cell metabolic pathways, the neurotrophin pathway and the MAPK signaling pathway (Supplementary Fig. S3C and Supplementary Table S4). Interestingly, shRNAs targeting genes that are involved in calcium signaling (e.g. P2RX1, PDE1C, TACR1, NOS3 and ITPR2), autophagy (e.g. ATG4A and ATG4C) and the estrogen signaling pathway (e.g. ITPR2, CREB3L2, NOS3 and SOS2) were found to be depleted in the surviving cells, suggesting that the inhibition of these pathways in combination with JA treatment may evoke a synergistic response (Supplementary Fig. S3C and Supplementary Table S5).

### Combined functional genomic and proteomic approaches identify the cancer spliceosome as a putative target of JA

Next we ‘intersected’ the proteomic data set with the RNAi screen data set. In the intersection, we found 28 targets that were required for JA activity and were induced in JA-treated cells (Fig. 2A and Supplementary Table S6). Among these targets was the Fas-associated *via* death domain (FADD) which is known to regulate apoptosis and necroptosis^30^, and glycogen synthase kinase 3 beta (GSK3B), which has been shown to function in a wide range of cellular processes and signaling pathways including suppression of the Wnt/beta-catenin pathway^31^. Functional enrichment analysis was carried out using the Kyoto Encyclopedia of Genes and Genomes (KEGG) database to classify the candidate targets into statistically significant over-represented functional categories. The genes involved in the spliceosome were significantly over-represented (P < 0.01, Fig. 2B). To further investigate the mechanism of action of JA, the 28 candidate targets were mapped onto protein-protein interaction networks using STRING^32^. Interestingly, the analysis revealed that 7 targets (SF3B1, SF3B3, SNRPA, SNRPE, SNRPF, HNRNPA3 and EFTUD2) are physically interacting proteins within the spliceosome complex, suggesting that JA might regulate the cancer spliceosome to induce tumor-specific cell death (Fig. 2C).

### JA triggers dysregulation of SF3B1 and SF3B3 proteins

**Figure 3:**
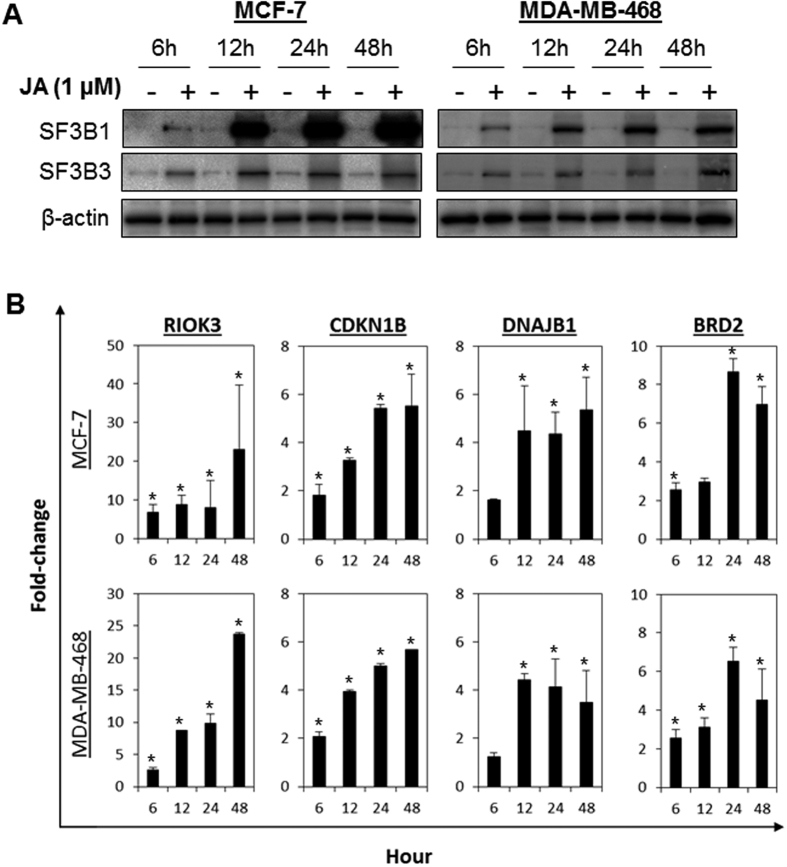
JA induces SF3B1 and SF3B3 protein expression and induces mRNA splicing errors. (**A**) MCF-7 and MDA-MB-468 cells were treated with 1 μM of JA for 6, 12, 24 and 48 h, followed by immunoblotting analyses. Increased accumulation of SF3B1 and SF3B3 was detected following treatment with JA. Cropped blot is shown from one representative experiment. Full-length gels are included in the Supplementary information file. (**B**) JA induced mRNA splicing errors in MCF-7 and MDA-MB-468 cells. Cells were treated with 1 μM of JA for 6, 12, 24 and 48 h. Unspliced pre-mRNA was quantified using qPCR targeting the intronic regions of RIOK3, CDKN1B, DNAJB1, and BRD2. Bars represent the means ± s.d. of at least 3 independent experiments. Asterisks (*) indicate statistical significance compared with vehicle cells (P < 0.01, Student’s *t*-test). Note the significant induction of protein and unspliced pre-mRNA in JA-treated cells as early as 6 h after treatment.

To validate whether JA regulates the cancer spliceosome, we carried out immunoblotting to confirm the expression of SF3B1 and SF3B3 following JA treatment in MCF-7 and MDA-MB-468 cells. As shown in Fig. 3A, JA induced a time-dependent increase in cellular SF3B1 and SF3B3 proteins, consistent with the results obtained from the MudPIT analysis. Of note, the induction of SF3B1 and SF3B3 was observed as early as 6 h after treatment, which corroborated with the induction of apoptosis (Fig. 1D). Interestingly, no significant changes in mRNA levels of the SF3B family in response to JA were detected (Supplementary Fig. S4A), suggesting that the induction of protein expression is independent of transcriptional activity. Further analyses after the cycloheximide chase assay revealed that JA markedly prolonged the life-span of the SF3B1 protein in both MCF-7 and MDA-MB-468 cells, suggesting that JA regulates SF3B1 protein stability rather than transcriptional activation (Supplementary Fig. S4B,C). In contrast to the breast cancer cells, both SF3B1 and SF3B3 were found to be down-regulated by JA in the non-transformed MCF-10A cells (Supplementary Fig. S5A).

### JA induces pre-mRNA splicing errors

As previous studies have demonstrated that anticancer spliceosome-targeting compounds such as pladienolides and sudemycin inhibit splicing activity^33^,^34^,^35^, we sought to evaluate the effect of JA on splicing activity by quantifying the amounts of unspliced pre-mRNA of RIOK3, CDKN1B, DNAJB1, and BRD2 by qPCR using primer sets specific for the intronic regions as described previously^33^,^35^. We observed that JA treatment resulted in a significant increase in unspliced pre-mRNA in MCF-7 and MDA-MB-468 cells, indicating that JA significantly impairs splicing activity (Fig. 3B and Supplementary Fig. S6A). In contrast, the amounts of unspliced pre-mRNA of RIOK3 and CDKN1B were significantly lower in MCF-10A cells treated with JA (Supplementary Fig. S5B), corroborating the down-regulation of SF3B1 and SF3B3 in these cells (Supplementary Fig. S5A). Of note, no significant changes in the levels of spliced mRNA was observed in cells following JA treatment (Supplemental Figs S5B and S6B).

### Induction of SF3B1 impairs pre-mRNA splicing and induces apoptosis in breast cancer cells

Since SF3B1 and SF3B3 have been shown to be a core component of the U2 snRNP of the spliceosome and is essential in regulating pre-mRNA splicing in cancer cells^4^, we hypothesize that the dysregulation of SF3B1 and/or SF3B3 (activation and inhibition) might result in the loss of critical proteins and the expression of variants with aberrant function that influence the survival of cancer cells.

**Figure 4:**
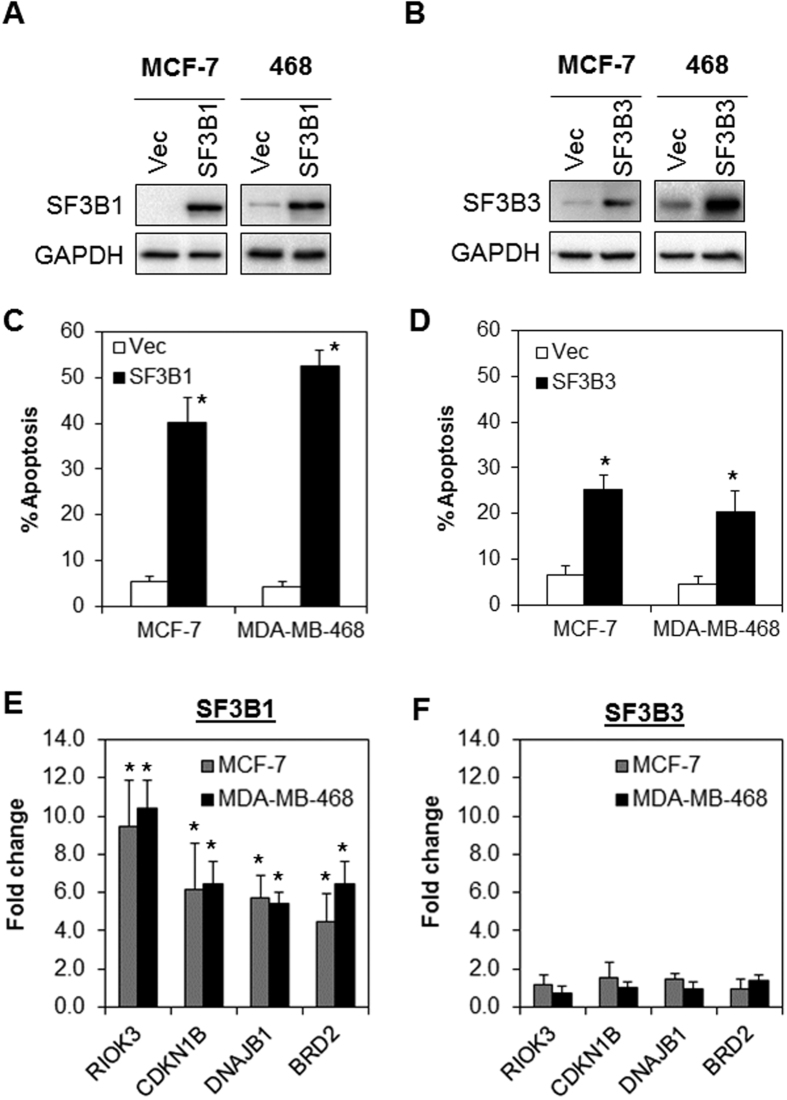
Overexpression of SF3B1 and SF3B3 induces splicing errors and tumor-specific apoptosis. (**A** and **B**) SF3B1 and SF3B3 were transiently overexpressed in MCF-7 and MDA-MB-468 cells. Cropped blot is shown from one representative experiment. Full-length gels are included in the Supplementary information file. (**C** and **D**) Ectopic SF3B1 and SF3B3 expression induced significant apoptosis. (**E** and **F**) Ectopic expression of SF3B1, but not SF3B3, induced unspliced RIOK3, CDKN1B, DNAJB1, and BRD2 pre-mRNA expression. Asterisks (*) indicate statistical significance compared with control cells transfected with the empty vector (P < 0.01, Student’s *t*-test).

To test whether SF3B1 and SF3B3 upregulation alone would trigger apoptosis and perturb splicing activity, SF3B1 and SF3B3 was transiently overexpressed in MCF-7 and MDA-MB-468 (Fig. 4A,B). Indeed, we observed that SF3B1 and SF3B3 overexpression induced significant apoptosis (Fig. 4C,D). Similarly, overexpression of SF3B1 also inhibited splicing activity in both cell lines, analogous to the phenotype induced by JA (Fig. 4E). In contrast, the splicing of RIOK3, CDKN1B, DNAJB1, and BRD2 remained unchanged in SF3B3 overexpressed cells (Fig. 4F).

### SF3B1 and SF3B3 are required for the antitumor effects of JA

**Figure 5:**
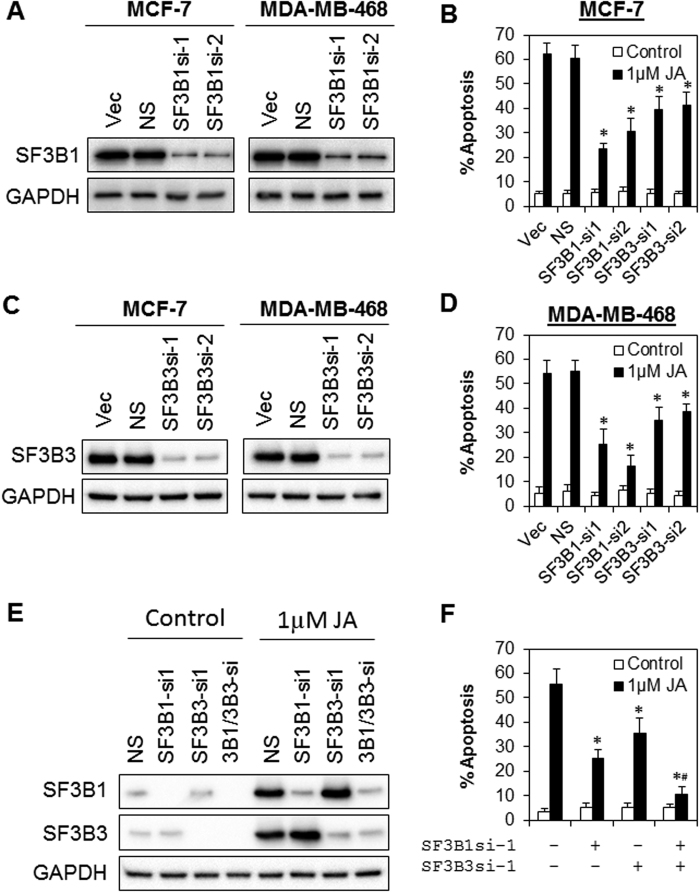
The antitumor activity of JA is mediated by SF3B1 and SF3B3. (**A–D**) A stable pool of isogenic cells depleted for SF3B1 or SF3B3 was generated in MCF-7 and MDA-MB-468 cells using two independent lentiviral shRNA constructs targeting the endogenous proteins, followed by brief puromycin selection. Levels of knock-down were evaluated by immunoblotting. SF3B1 or SF3B3 depleted cells were treated with 1 μM of JA. Apoptosis was evaluated at 72 h after treatment. Cropped blot is shown from one representative experiment. Full-length gels are included in the Supplementary information file. Bars represent the means ± s.d. of 3 independent experiments. Asterisks (*) indicate statistical significance compared with control cells without JA treatment (P < 0.01, Student’s *t*-test). Vec, vector control cells. NS, non-targeting shRNA. (**E** and **F**) Simultaneous knock-down of SF3B1 and SF3B3 completely abrogated the apoptotic effects of JA. MCF-7 cells were transfected with SF3B1si-1 and/or SF3B3si-1 for 48 h followed by JA treatment for 24 h. Cropped blot is shown from one representative experiment. Full-length gels are included in the Supplementary information file. Asterisks (*) indicate statistical significance compared with control cells without JA treatment (P < 0.01, Student’s *t*-test). Hash (#) indicates statistical significance compared to SF3B1- or SF3B3-depleted cells following treatment with JA (P < 0.01, Student’s *t*-test).

Next, we investigated whether SF3B1 or SF3B3 mediate the antitumor activity of JA. We generated a stable pool of isogenic cells depleted for SF3B1 or SF3B3 by the transduction of two independent lentiviral shRNA constructs targeting the endogenous proteins in MCF-7 and MDA-MB-468 cells, followed by brief drug selection with puromycin. Indeed, depletion of SF3B1 or SF3B3 partially rescued the apoptotic cell death induced by JA in both MCF-7 and MDA-MB-468 cells (Fig. 5A–D). Furthermore, double knock-down of SF3B1 and SF3B3 completely abrogated the apoptotic effects of JA, suggesting that both SF3B1 and SF3B3 are required for the antitumor effects of JA (Fig. 5E,F). Also, knock-down of SF3B1, but not SF3B3, significantly reduced the accumulation of unspliced pre-mRNA induced by JA (Supplementary Fig. S7), suggesting that the mis-splicing of RIOK3, CDKN1B, BRD2 or DNAJB1 following JA treatment is dependent on SF3B1 but not SF3B3. SF3B1 depletion, however, did not rescue the G2/M cell cycle arrest induced by JA treatment (Supplementary Fig. S8), indicating that SF3B1 is dispensable for the cytostatic effects of JA, but more critical for apoptosis execution.

### JA disrupts the interactions of SF3B1 and SF3B3 in the nucleosome complexes

**Figure 6:**
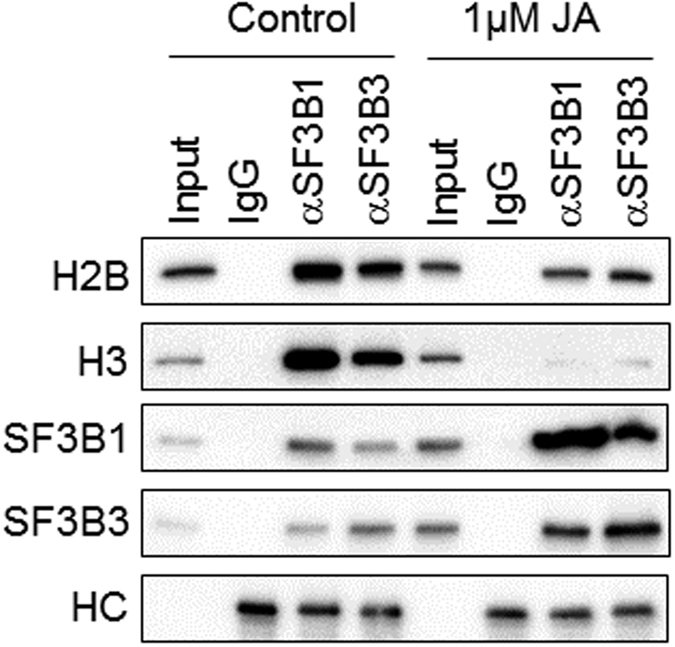
JA disrupts the interactions of SF3B1 and SF3B3 with histone H2B and H3 in the nucleosome complexes. MCF-7 cells were treated with 1 μM of JA for 24 h. Immunoprecipitation was performed with antibodies against SF3B1, SF3B3 or non-specific IgG. Western blotting was performed with the indicated antibodies. The heavy chain (HC) of the IgG served as loading control. Cropped blot is shown from one representative experiment. Full-length gels are included in the Supplementary information file.

A recent study has shown that SF3B1 is associated with the nucleosome complexes and affects splicing outcomes^36^. To evaluate whether the association of SF3B1 and SF3B3 in the nucleosome complexes might be affected by JA, we performed co-immunoprecipitation (co-IP) assays in MCF-7 cells treated with JA for 24 h. Our results indicated a strong association of SF3B1 and SF3B3 with H2B and H3 in the nucleosome (Fig. 6). However, such associations were diminished following treatment of cells with JA. These results suggest that JA disrupts the interactions of SF3B1 and SF3B3 with the nucleosome. Together, our results demonstrated that JA exerts its antitumor activity in cancer cells at least in part by targeting SFB1 (and SFB3).

## Discussion

The spliceosome represents an under-explored and unusual target in cancer cells. The recent identification of small molecules (e.g. Spliceostatin, Sudemycins and Pladienolides) that interact with SF3B1, and the identification of mutant SF3B1 in tumor samples indicate that this complex can be a viable target for chemotherapeutic intervention^2^,^3^. While the exact mechanism of cytotoxicity induced by these spliceosome modulators is currently unclear, evidence indicates that interaction with SF3B1 is crucial for their antitumor activity^2^,^3^.

JA is an anticancer compound that has been previously reported by our group to possess remarkable antitumor activities^27^,^28^. Using combined proteomic and functional genomics analyses, we identified SF3B1 and SF3B3 as putative targets of JA. However, unlike other spliceosome modulators which have been shown to inhibit SF3B1, JA was found to induce SF3B1 and SF3B3 protein expression through increased protein stability. Intriguingly, the induction of SF3B1 by JA or ectopic expression of SF3B1 in MCF-7 and MDA-MB-468 cells impairs mRNA splicing activity as evidenced by the accumulation of unspliced RIOK3, CDKN1B, DNAJB1 and BRD2 pre-mRNA. In contrast, the amounts of unspliced pre-mRNA remained unchanged following ectopic expression of SF3B3 in MCF-7 and MDA-MB-468 cells, despite observation of significant apoptotic cell death. Of note, depletion of the endogenous SF3B1 or SF3B3 partially inhibited the apoptosis induced by JA in breast cancer cells, while the simultaneous knock-down of SF3B1 and SF3B3 completely abrogated the apoptotic effects of JA, suggesting that both SF3B1 and SF3B3 are required for the antitumor activity of JA in breast cancer cells. However, only knock-down of SF3B1, but not SF3B3, significantly reduced the accumulation of unspliced pre-mRNA following JA treatment indicating that the mis-splicing of RIOK3, CDKN1B, BRD2 or DNAJB1 following JA treatment is dependent on SF3B1 but not SF3B3. These results suggest that SF3B1 and SF3B3 might induce tumor specific cell death through distinct mechanisms. Indeed, SF3B3 has been recently shown to regulate alternative splicing of EZH2 pre-mRNA in renal cancer and mediates ubiquitination through modulation of the NEDD8 pathway^37^,^38^.

Numerous studies have also demonstrated that overexpression and mutations of key spliceosome components are associated with malignancies in various tissues, resulting in aberrant splicing activity and the emergence of hallmark cancer phenotypes^3^,^6^,^23^,^39^,^40^,^41^. Alterations in SF3B1 have been reported in myelodysplastic syndromes (~20%)^19^,^42^,^43^, acute myeloid leukemia (~5%)^19^, chronic lymphocytic leukemia (~5%)^12^,^19^ and other solid tumors including breast cancer (~1%)^19^,^20^,^21^, renal cancer (~3%)^19^ and adenoid cystic carcinoma (~4%)^19^. In breast cancer, in particular, SF3B1 mutations were significantly associated with ER-positive disease, *AKT1* mutations, and distinct copy number alterations^20^. While the potential function of SF3B1 mutants has yet to be fully elucidated, the spliceosome modulator, spliceostatin A, was found to be more potent in cell lines harboring SF3B1 mutations compared to SF3B1 wild-type cells^20^. This, however, was not observed in the JA-treated cells, as the SF3B1 wild-type MDA-MB-468, T47D and MCF7 cells are equally sensitive to JA as compared to the HCC38 cells, which harbors a heterozygous SF3B1 Q534P missense mutation (data not shown)^44^. This result suggests that the antitumor effect of JA is independent of SF3B1 mutations.

It is intriguing to investigate how the dysregulation of SF3B1, via either activation or inhibition, could lead to tumor-specific cell death. It is known that the maintenance of high-fidelity mRNA splicing is important, because the translation of mis-spliced mRNAs into proteins with aberrant function could be deleterious to all cells. Therefore, it is not surprising that eukaryotic cells have evolved a protective mechanism, known as nonsense-mediated decay (NMD), to target and eliminate the inappropriately spliced mRNAs^45^. Interestingly, several pathways involved in NMD were recently found to be inactivated in many cancers including breast, colon, liver, lung, thyroid and esophagus cancer^46^. Although it is currently unclear whether JA sensitivity is dependent on NMD deficiency, a recent study showed that NMD efficiency in MCF-7 cells, which are sensitive to JA, was significantly lower compared to HeLa cells^47^. Similarly, siRNA-mediated depletion of SNRPE or SNRPD1, components of the core spliceosomal heteroheptameric Sm complex, also led to a marked reduction of cell viability in MDA-MB-468 and SKBR3 breast cancer cells, but not in the non-tumoral MCF-10A cells^48^. Thus, it is plausible that the dysregulation of SF3B1 and spliceosome activity by JA might lead to an imbalance in the splicing program in susceptible cancer cells, which may induce apoptosis by the accumulation of mis-spliced mRNAs into deleterious proteins.

While most spliceosome modulators vary greatly in terms of chemical structure, they have all been shown to have almost identical effects on spliceosome assembly^49^,^50^. A recent study has demonstrated that spliceostatin A, pladienolide B and herboxidiene all interact with the same site on SF3B1, and likely exhibit the same mechanism of action of inhibiting spliceosome assembly, possibly by interfering with SF3B1 conformational states^50^. Also, unlike the splicing modulator sudemycin which was recently shown to induce a specific antitumor response in chronic lymphocytic leukemia through alternative splicing of MCL1 to generate its proapoptotic isoform^51^, no such effect was observed in MCF-7 and MDA-MB-468 treated with JA (Supplementary Fig. S9). Whether JA interacts with SF3B1 in a similar manner to that of the aforementioned spliceosome modulators remains to be further investigated. However, based on the distinct mechanism of JA in stabilizing SF3B1, rather than inhibition as demonstrated by other spliceosome modulators, it is likely that JA might engage a novel mechanism to regulate the cancer spliceosome activities. Hence, further understanding of how JA might interact with SF3B1 will be invaluable for the design and further development of new drug candidates.

In conclusion, our findings indicate that JA evokes tumor-specific activity by targeting SF3B1 and causing abnormal splicing patterns, in addition to its previously reported activity of inhibiting microtubule polymerization. While the precise mechanism by which JA inhibits splicing activity has yet to be elucidated, the fact that it is multi-targeted as well as tumor-specific makes it an attractive candidate for further development. More comprehensive understanding of these mechanisms, coupled with knowledge of the spliceosome functions in tumors are important for the further development of this novel class of experimental antitumor agents.

## Methods

### Chemicals and reagents

Jerantinine A was isolated from the leaf ethanolic extract of the Malayan *Tabernaemontana corymbosa* Roxb. ex Wall and characterized as reported previously^25^.

### Cell lines and cell culture

The human breast cancer cell lines (MDA-MB-468, T47D and MCF7) were obtained from American Type Culture Collection (ATCC) and were maintained in RPMI-1640 medium containing 10% fetal bovine serum (FBS), 100 IU/ml of penicillin, and 100 μg/ml of streptomycin (Sigma-Aldrich, St. Louis, MO, USA). The non-transformed human mammary epithelial cell line MCF-10A was cultured in DMEM/F12 (Invitrogen, Carlsbad, CA, USA) supplemented with 5% horse serum, 20 ng/ml epidermal growth factor (EGF), 0.5 μg/ml hydrocortisone, 10 μg/ml insulin, 100 IU/ml penicillin, and 100 μg/ml streptomycin. All cells were maintained at 37 °C in 5% CO_2_. Cells were passaged for less than 6 months and no further authentication was performed by the authors.

### Cell proliferation assay

Dose-response curves and IC_50_ values were determined using the 3-(4,5-dimethylthiazol-2-yl)-2,5-diphenyltetrazolium bromide (MTT) cell proliferation assay, as described previously^52^,^53^.

### Flow cytometric detection of apoptosis

The population of apoptotic cells was quantified using the PE Annexin V Apoptosis Detection Kit (BD Biosciences, San Jose, CA, USA), according to the manufacturer’s instructions. Both floating and attached cells were collected, stained, and analyzed using a FACSCalibur flow cytometer and the CellQuest Pro software (version 5.1.1; BD Biosciences, USA).

### Proteomic profiling

MCF-7 cells were treated with vehicle or 1 μM of JA for 8 h. Cells were harvested using a cell lifter and washed twice with phosphate buffer saline (PBS). LC–MS/MS analysis was performed as a service by Bioproximity LLC (Chantilly, VA, USA).

### Pooled genome-wide shRNA library screen

MCF-7 cells were transduced with MISSION LentiPlex Human Pooled shRNA Library, targeting more than 15000 human genes (Sigma-Aldrich, USA). Tranduced cells were selected with 1 μg/mL puromycin for 4 days and treated with vehicle or 1 μM of JA for 72 h. Genomic DNA was isolated using the GenElute Mammalian Genomic DNA Miniprep Kit (Sigma, USA) according to the manufacturer’s protocol. shRNA sequences present in each sample were determined by next-generation sequencing (NGS). Massively parallel sequencing for shRNA retrieval and screen data analysis were described in Supplementary Methods.

### Quantitative real-time PCR analysis

mRNA expression levels were measured by real-time quantitative PCR (qPCR) as described previously^54^,^55^. Briefly, total cellular RNA was extracted using the Qiagen RNA Isolation Kit (Qiagen, Valencia, CA, USA). First-strand cDNA was synthesized using the High Capacity RNA-to-cDNA Master Mix (Applied Biosystems, Foster City, CA, USA) according to the manufacturer’s instructions. Gene expression levels were measured by qPCR using the FastStart Universal SYBR Green Master (Roche Diagnostics, Indianapolis, IN, USA) and a Biorad iQ5 real-time PCR detector system (Bio-Rad, Hercules, CA, USA). Spliceosome activity was estimated by amplification of the intronic regions of DNAJB1, CDKN1B, RIOK3 and BRD2, as described previously^51^,^56^,^57^. qPCR conditions for all amplifications are as follows: 3 min at 94 °C followed by 40 cycles of 94 °C for 40 sec, 60 °C for 40 sec, and 72 °C for 25 sec. Gene expression data were normalized to GAPDH as the house-keeping gene. The specific primers are shown in Supplementary Table S7.

### Transfection

The SF3B1 and SF3B3 expression construct was obtained from OriGene Technologies (Rockville, MD, USA). Transient transfections were performed using X-tremeGENE HP DNA transfection reagent (Roche Diagnostics, USA) according to the manufacturer’s instructions.

### Lentivirus production and transduction

Lentiviral non-targeting shRNA (NS) and shRNA constructs targeting SF3B1 and SF3B3 were purchased from Sigma-Aldrich (St. Louis, MO, USA). The shRNAs target sequences were presented in Supplementary Table S8. Lentivirus production and transduction were performed as described previously^52^,^54^. Briefly, high-titer lentiviral stocks were generated by co-transfection with packaging plasmids psPAX2 (Addgene plasmid #12260) and envelope plasmids pMD2.G (Addgene plasmid #12259) into HEK-293T using CalPhos Transfection Kits (Clontech, Mountain View, CA, USA). Supernatants containing lentiviral stocks were supplemented with polybrene (Sigma, USA) and used for transduction of target cells. Stable pools were generated by transduction of two independent lentiviral shRNA constructs targeting SF3B1 followed by brief selection with puromycin (Sigma, USA).

### Protein isolation and immunoblotting

Protein lysates were extracted using ice-cold lysis buffer (1% NP-40, 1 mM DTT, supplemented with protease and phosphatase inhibitors in PBS). Total protein (50 μg) was subjected to SDS-PAGE followed by immunoblotting. Primary antibodies against SF3B1 and SF3B3 were obtained from Antibodies-online GmbH (Aachen, Germany). Mouse monoclonal antibodies against β-actin (clone C-2; 1:250) and GAPDH (G-9; 1:1,000) were obtained from Santa Cruz Biotechnology (Dallas, Texas, USA). Rabbit monoclonal antibodies against histone H2B and histone H3 were purchased from Cell Signaling Technology (Danvers, MA, USA).

## Additional Information

**How to cite this article**: Chung, F. F.-L. *et al*. Jerantinine A induces tumor-specific cell death through modulation of splicing factor 3b subunit 1 (SF3B1). *Sci. Rep.* **7**, 42504; doi: 10.1038/srep42504 (2017).

**Publisher's note:** Springer Nature remains neutral with regard to jurisdictional claims in published maps and institutional affiliations.

## Supplementary Material

Supplementary Information
