# Supplementary material for: Jerantinine A induces tumor-specific cell death through modulation of splicing factor 3b subunit 1 (SF3B1)
Source: Sci Rep. 2017 Feb 15;7:42504. doi: 10.1038/srep42504 (PMC5309811; doi:10.1038/srep42504)

**Jerantinine A induces tumor-specific cell death through modulation of splicing factor 3b subunit 1 (SF3B1)**

Felicia Fei-Lei Chung^1^, Perry Faith Tze Ming Tan^2^, Vijay Joseph Raja^3^, Boon-Shing Tan^4^, Kuan-Hon Lim^5^, Toh-Seok Kam^6^, Ling-Wei Hii^1,7^, Si Hoey Tan^1,7^, Sze-Jia See^1^, Yuen-Fen Tan^1,7^, Li-Zhe Wong^1,7^, Wai Keat Yam^8^, Chun Wai Mai^8^, Tracey D. Bradshaw^9^, Chee-Onn Leong^1,8*^

^1^ Center for Cancer and Stem Cell Research, International Medical University, Bukit Jalil, 57000 Kuala Lumpur, Malaysia.

^2^ School of Medicine, International Medical University, Bukit Jalil, 57000 Kuala Lumpur, Malaysia.

^3^ Department of Biochemistry, Weill Cornell Medical College, New York, NY 10021, USA.

^4^ Institute of Biological Chemistry, Academia Sinica, Taipei, Taiwan

^5^ School of Pharmacy, University of Nottingham Malaysia Campus, Jalan Broga, 43500 Semenyih, Selangor, Malaysia.

^6^ Department of Chemistry, Faculty of Science, University of Malaya, 50603 Kuala Lumpur, Malaysia

^7^ School of Postgraduate Studies, International Medical University, Bukit Jalil, 57000 Kuala Lumpur, Malaysia.

^8^ School of Pharmacy, International Medical University, Bukit Jalil, 57000 Kuala Lumpur, Malaysia.

^9^ School of Pharmacy, University of Nottingham, University Park, Nottingham NG7 2RD, UK

* Corresponding author: [cheeonn_leong@imu.edu.my](mailto:cheeonn_leong@imu.edu.my) (COL)

**SUPPLEMENTARY METHODS**

**Massively parallel sequencing and shRNA screen data analysis pipeline**

shRNA sequences integrated into the genomic DNA of the screening cells were recovered by PCR amplification using construct-specific PCR primers with DNA barcode tails unique to each sample as described previously [^1-3^](#_ENREF_1). Purified PCR products from each sample were pooled into one tube. An Illumina sequencing library was generated using the barcoded and pooled PCR products. The pool of PCR products (and barcodes for each sample) were sequenced on the Illumina platform generating millions of sequence reads. Sequence reads were binned into sample specific groups based on the unique barcode sequence incorporated during the PCR step. Each bin was analysed separately by aligning to a shRNA FASTA reference. Statistical analysis of screen results was performed in R using shRNA-seq [^1^](#_ENREF_1). Briefly, read counts per shRNA in each sample were log_2_ transformed and then the ratio of reads in JA-treated vs. vehicle treated samples was calculated. Each log ratio was then normalised to the average shRNA abundance in each pool using non-linear regression, to account for biases in shRNA abundance. Normalised log ratios were then re-scaled by the pool Median Absolute Deviation (MAD) to ensure comparable distributions between different pools. The resultant Drug Effect (DE) log ratios were then quantile normalized to allow comparison between biological replica screens. Hit identification detection was performed using three parallel methods. In the first method, a Z-score threshold of > 2 or < -2, and were targeted by at least two independent shRNAs were considered as screen hits. As alternative methods, RSA and RIGER were also used as described previously . Subset of the genes identified by all three methods was defined as candidate genes and subject for further examination.

**Bioinformatics and network mapping**

The DAVID Bioinformatics resource 6.7 and the ConsensusPathDB-human (CPDB) Release 31 was used to generate functional annotation gene lists for candidate genes based on curated biological pathways (e.g. KEGG) [^6-9^](#_ENREF_6). STRING v10.0 was used to map candidate genes onto protein-protein interaction networks ([http://string-db.org](http://string-db.org/)) [^10^](#_ENREF_10). The highest confidence threshold (0.9) was used to define protein-protein interactions.

### Caspase activity assay

Cells were seeded in 96-well plates overnight at a density of 3 x 10^3^ cells per well and treated with 1 µM of JA. Caspase catalytic activity of caspase 3/7, caspase 8 and caspase 9 were determined at 6, 12, 24 and 48 h after treatment using Caspase-Glo 3/7, Gaspase-Glo 8, and Caspase-Glo 9 Assay kits (Promega, Madison, WI, USA) according to the manufacturer’s instructions.

**Protein stability assay**

MCF-7 cells were pre-treated with 50 μg/mL of cycloheximide (CHX, Cayman Chemicals, Ann Arbor, Michigan, USA) for 1 h followed by 1µM of jerantinine A or vehicle at the indicated time points. Samples were analyzed by western blotting for SF3B1 degradation. Protein densitometry was analysed using the Bio-Rad Image Lab ver. 4.1, build 16 (Bio-Rad, USA).

**REFERENCES**

1 Sims, D. *et al.* High-throughput RNA interference screening using pooled shRNA libraries and next generation sequencing. *Genome Biol* **12**, R104, doi:10.1186/gb-2011-12-10-r104 (2011).

2 Zuber, J. *et al.* Toolkit for evaluating genes required for proliferation and survival using tetracycline-regulated RNAi. *Nat Biotechnol* **29**, 79-83, doi:10.1038/nbt.1720 (2011).

3 Silva, J. M. *et al.* Profiling essential genes in human mammary cells by multiplex RNAi screening. *Science* **319**, 617-620, doi:10.1126/science.1149185 (2008).

4 Subramanian, A. *et al.* Gene set enrichment analysis: a knowledge-based approach for interpreting genome-wide expression profiles. *Proc Natl Acad Sci U S A* **102**, 15545-15550, doi:0506580102 [pii] 10.1073/pnas.0506580102 (2005).

5 Luo, B. *et al.* Highly parallel identification of essential genes in cancer cells. *Proc Natl Acad Sci U S A* **105**, 20380-20385, doi:10.1073/pnas.0810485105 (2008).

6 Huang da, W., Sherman, B. T. & Lempicki, R. A. Systematic and integrative analysis of large gene lists using DAVID bioinformatics resources. *Nat Protoc* **4**, 44-57, doi:10.1038/nprot.2008.211 (2009).

7 Kamburov, A., Stelzl, U., Lehrach, H. & Herwig, R. The ConsensusPathDB interaction database: 2013 update. *Nucleic Acids Res* **41**, D793-800, doi:10.1093/nar/gks1055 (2013).

8 Kamburov, A., Wierling, C., Lehrach, H. & Herwig, R. ConsensusPathDB--a database for integrating human functional interaction networks. *Nucleic Acids Res* **37**, D623-628, doi:10.1093/nar/gkn698 (2009).

9 Kamburov, A. *et al.* ConsensusPathDB: toward a more complete picture of cell biology. *Nucleic Acids Res* **39**, D712-717, doi:10.1093/nar/gkq1156 (2011).

10 Szklarczyk, D. *et al.* The STRING database in 2011: functional interaction networks of proteins, globally integrated and scored. *Nucleic Acids Res* **39**, D561-568, doi:10.1093/nar/gkq973 (2011).

11 Xargay-Torrent, S. *et al.* The splicing modulator sudemycin induces a specific antitumor response and cooperates with ibrutinib in chronic lymphocytic leukemia. *Oncotarget* **6**, 22734-22749, doi:10.18632/oncotarget.4212 (2015).

**SUPPLEMENTAL FIGURES**


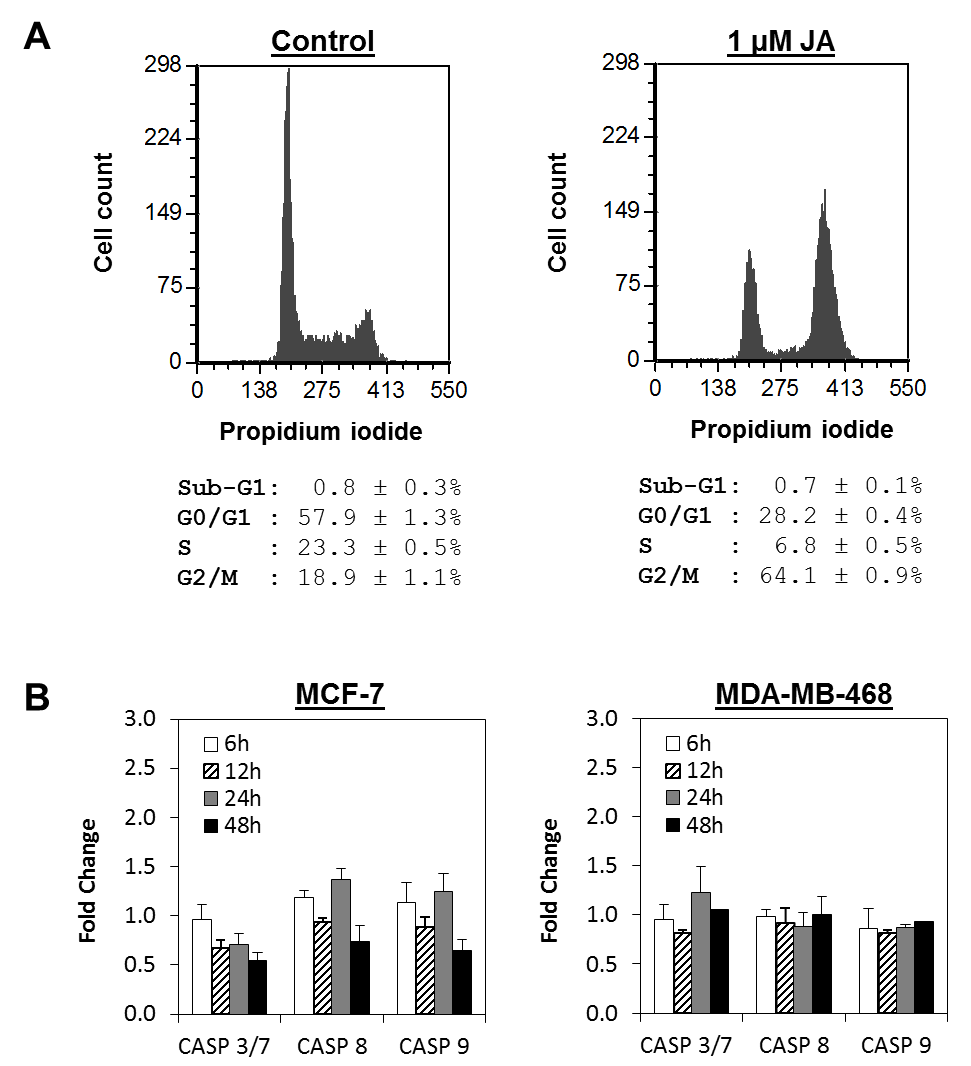


**Supplementary Figure S1: JA induced G2/M arrest and caspase-independent cell death. (A)** JA induces significant G2/M arrest in MCF-7 cells. Cells were treated with 1 µM of JA for 12 h followed by cell cycle analysis. Values represent the means ± s.d. of 3 independent experiments. Asterisks (*) indicate statistical significance compared with vehicle cells (P < 0.01, Student’s *t*-test). **(B)** No significant induction of caspase 3/7, 8 and 9 activity was observed in MCF-7 and MDA-MB-468 cells following treatment of 1 µM of JA. Bars represent the means ± s.d. of 3 independent experiments.


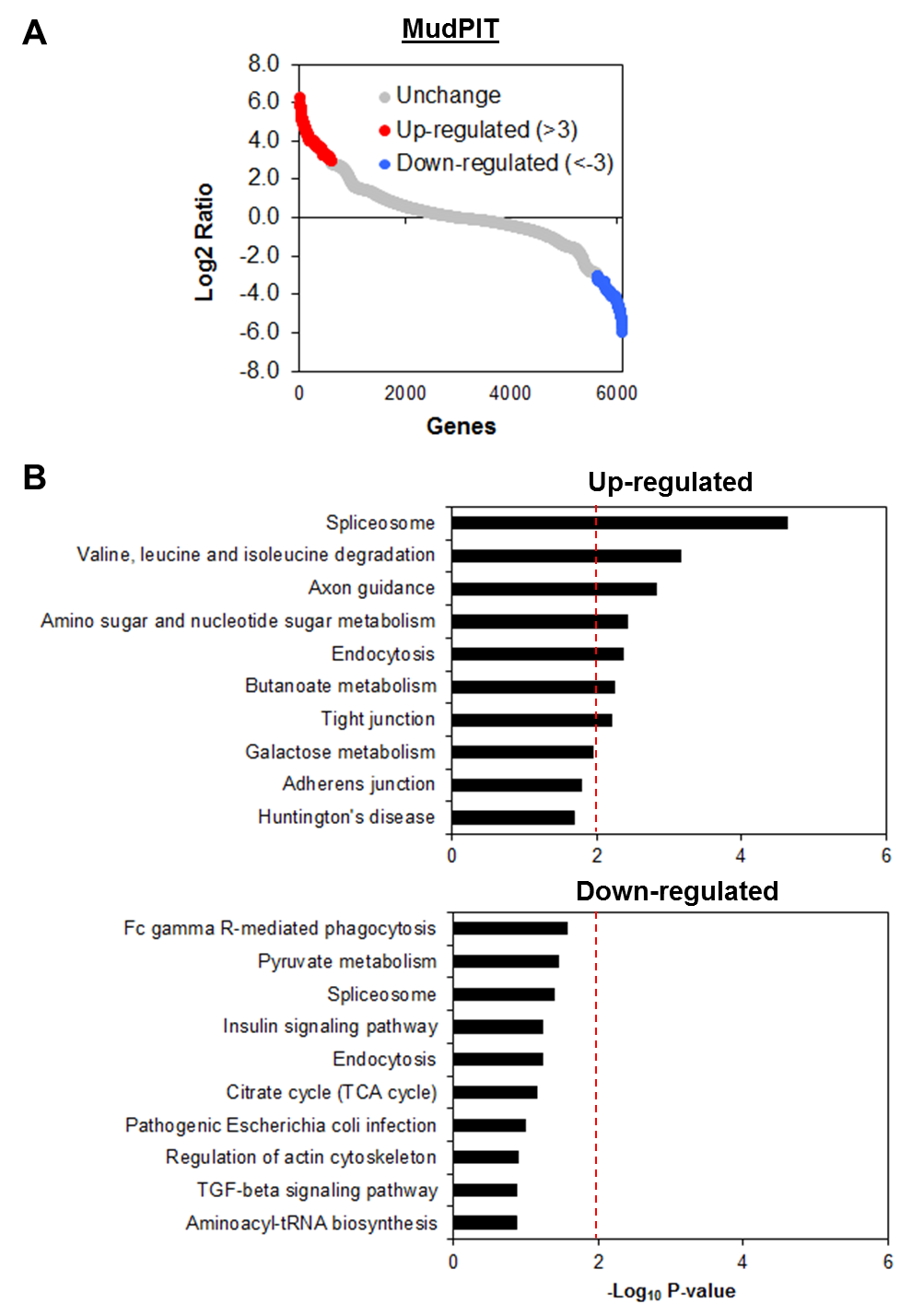


**Supplementary Figure S2: Proteomic profiling of MCF-7 breast cancer cells following JA treatment.** **(A)** Differential protein expression following JA treatment in MCF-7 cells. Rank order distribution of the data from MudPIT analysis. Unique peptide fragments were mapped to their corresponding genes using Proteome Cluster (see Supplementary Methods). Data are represented as Log_2_ ratio, with cut-offs of 3 and -3 designated as up-regulated (red, Supplementary Table 3) and down-regulated (blue, Supplementary Table 4) hits, respectively. **(B)** Pathway enrichment analysis for genes identified from MudPIT using CPDB. Red line indicates P < 0.01.


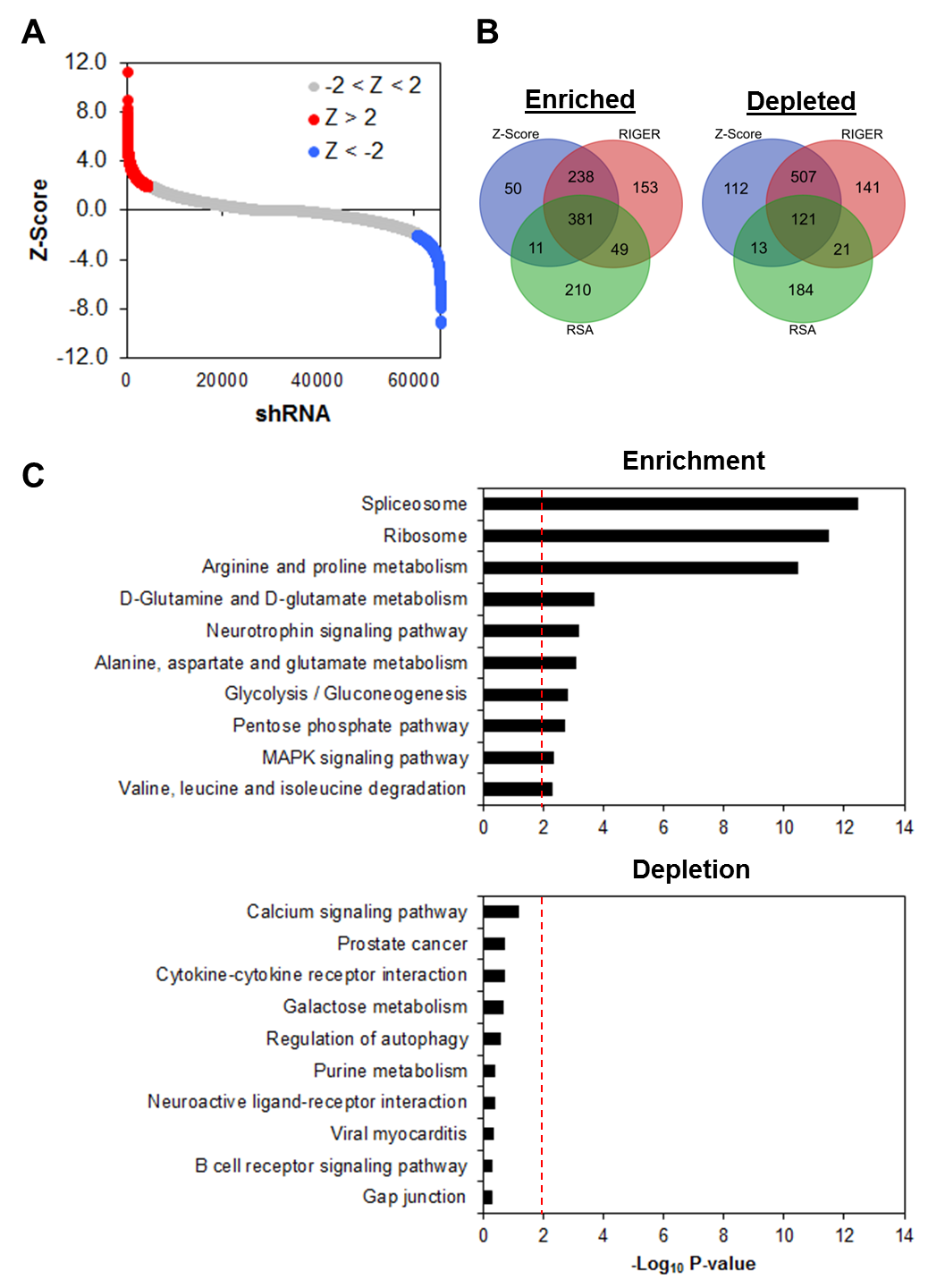


**Supplementary Figure S3: Genome-wide shRNA functional screen identifies a compendium of genes affecting sensitivity to JA. (A)** Plot of shRNA Z-scores ranked by size of effect. **(B)** Venn diagrams indicating the number of candidate hits defined by Z-scores, RIGER and RSA analyses (see Supplementary Methods). **(C)** Pathway enrichment analysis for genes identified from RNAi-screen using CPDB. Red line indicates P < 0.01.


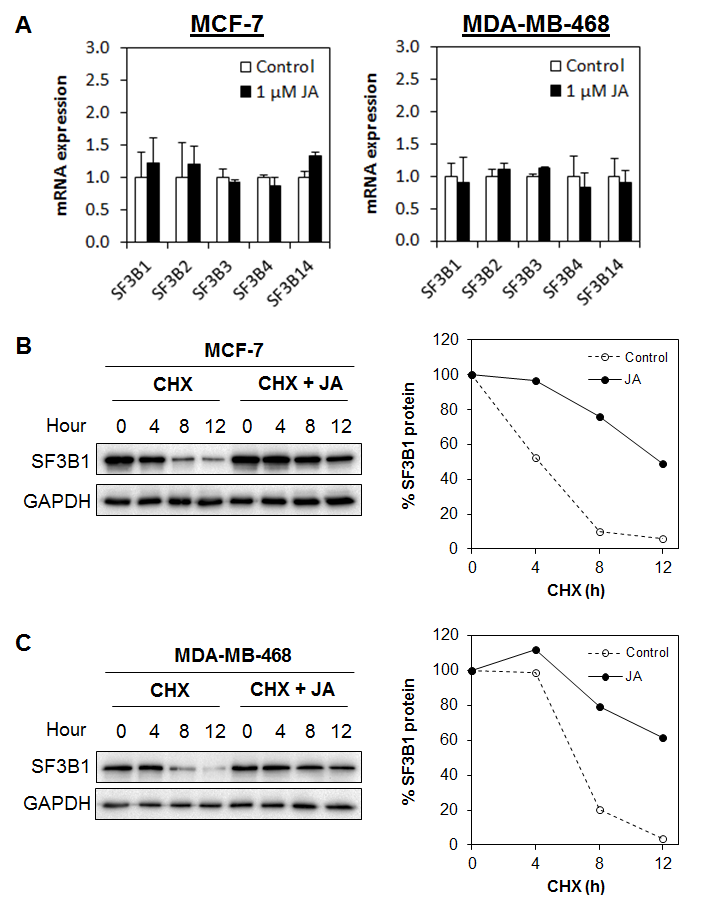


**Supplementary Figure S4: JA stabilizes SF3B1 proteins in MCF-7 and MDA-MB-468** **cells independent of SF3B1 mRNA transcription.** **(A)** No significant induction of SF3B1 mRNA was detected in response to JA treatment. mRNA expression of SF3B family (SF3B1, SF3B2, SF3B3, SF3B4 and SF3B14) were measured using qPCR at 24 h after JA-treatment. **(B)** JA prolonged the life-span of SF3B1 protein in both MCF-7 and MDA-MB-468 cells. Cells were pre-treated with 50 μg/mL of cycloheximide for 1 h followed by 1 µM of JA or vehicle at the indicated time points. The SF3B1 protein levels were plotted after normalization with GAPDH.


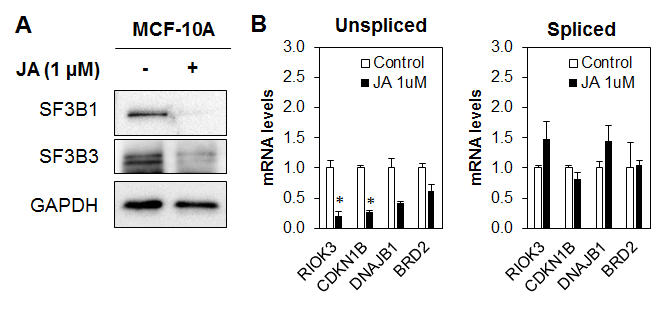


**Supplementary Figure S5: JA inhibits SF3B1 and SF3B3 protein expression in non-transformed MCF-10A cells. (A)** MCF-10Acells were treated with 1 µM of JA for 24 h followed by immunoblotting analyses. **(B)** JA suppressed mRNA splicing errors in MCF-10A. Cells were treated with 1 µM of JA for 24 h. Unspliced pre-mRNA was quantified using qPCR targeting the intronic regions of RIOK3, CDKN1B, DNAJB1, and BRD2. Spliced mRNA was quantified using qPCR targeting the exon/exon junction of the same genes. Bars represent the means ± s.d. of at least 3 independent experiments. Asterisks (*) indicate statistical significance compared with vehicle cells (P < 0.01, Student’s t-test).


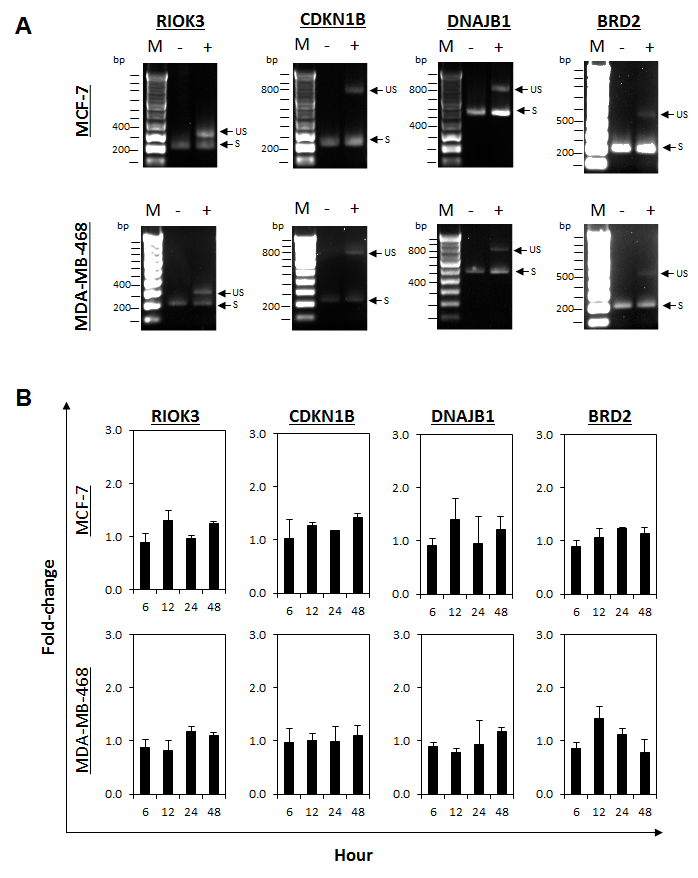


**Supplementary Figure S6: JA induces mRNA splicing errors. (A)** MCF-7 and MDA-MB-468 cells were treated with 1 µM of JA for 24 h followed by conventional end-point PCR spanning 2 exons. US, unspliced mRNA; S, spliced mRNA; M, markers; (-) control; (+) 1 µM of JA for 24 h. **(B)** Quantitation of spliced mRNA targeting the exon/exon junction of RIOK3, CDKN1B, DNAJB1, and BRD2. Bars represent the means ± s.d. of at least 3 independent experiments. Note, no significant different in the expression of spliced mRNA in JA-treated cells.


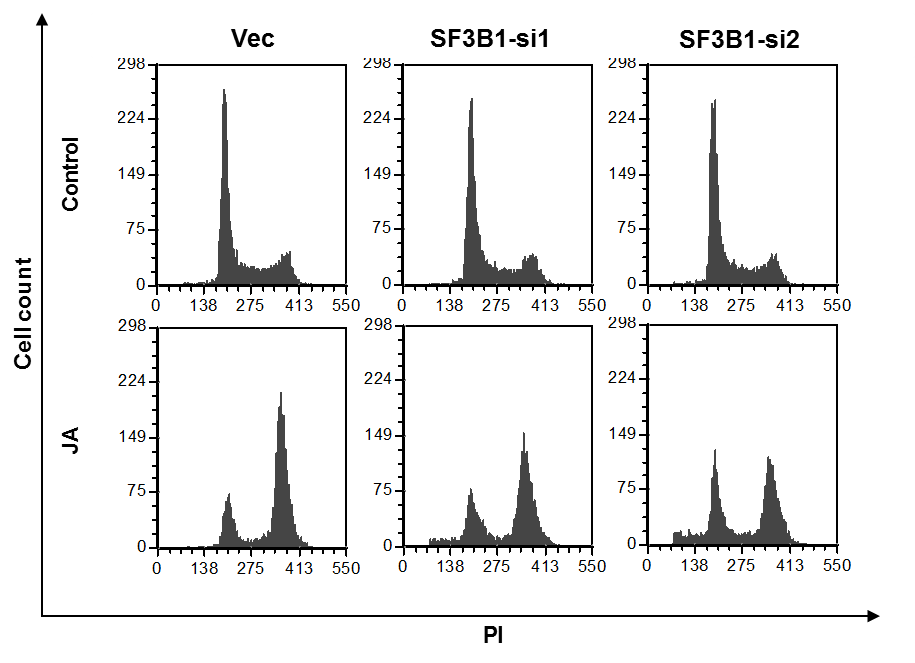


**Supplementary Figure S7: Induction of G2/M arrest by JA is independent of SF3B1 function.** SF3B1-depleted MCF-7 cells were treated with 1 µM of JA for 12 h followed by cell cycle analysis.


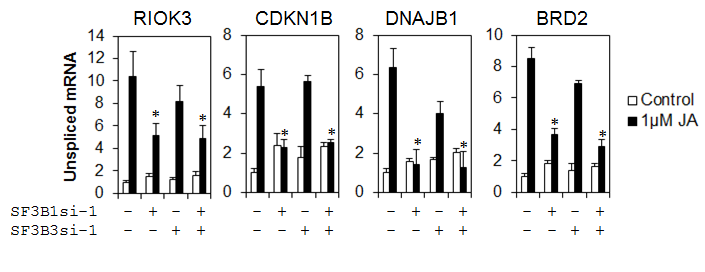


**Supplementary Figure S8: Depletion of SF3B1, but not SF3B3, reduced the levels of unspliced pre-mRNA in JA treated MCF-7 cells.** Cells were co-transfected with non-targeting shRNA, SF3B1si-1 and/or SF3B3si-1 for 48 h followed by JA treatment for 24 h as described in Figure 5E and F. Bars represent the means ± s.d. of at least 3 independent experiments. Asterisks (*) indicates statistical significance compared to vector control cells cells following treatment with JA (P < 0.01, Student’s t-test).


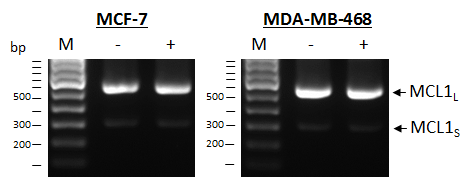


**Supplementary Figure S9: Effects of JA on the expression and alternative splicing of MCL1.** MCF-7 and MDA-MB-468 cells were treated with 1 µM of JA for 24 h and their mRNA isolated for PCR. The primer pair for or amplifying MCL1 was 5′- GAGGAGGAGGAGGACGAGTT-3′ and 5′-ACCAGCTCCTACTCCAGCAA-3′ as according to previous study[^11^](#_ENREF_11). The expected amplicon size for the long (MCL1_L_) and short (MCL1_S_) variants of the MCL1 are 531bp and 283bp, respectively. M, markers; (-) control; (+) 1 µM of JA for 24 h.

**SUPPLEMENTARY TABLES**

**Supplementary Table S1: IC_50_** values of JA breast cell lines.

| **Cell Lines** | **IC_50_ (µM)** |
| --- | --- |
| T47D | 0.72 ± 0.18 |
| MDA-MB-468 | 0.86 ± 0.09 |
| MCF-7 | 1.22 ± 0.15 |
| MCF-10A | >10 |

**Supplementary Table S2: List of proteins that were up-regulated in MCF-7 following JA treatment (log_2_ ratio of treatment/control > 3)**

14-3-3 protein

ABCE1

ABCF3

ABR

ACAD9

ACBD3

ACSF2

AFG3L2

AGR3

AIP

ALDH7A1

ALDH9A1

AMIGO2

ANAPC7

ANKFY1

ANKRD52

ANP32E

AP1G2

AP1S1

AP2M1

APPL1

ARCN1

ARFGAP2

ARL6IP5

ARMC6

ARPP19

ATP4A

ATP6V1B2

BAG6

BASP1

BCAT2

BCLAF1

BIRC6

BRIX1

BSG

BXDC2

C12orf57

C14orf142

C7orf55-LUC7L2

CA2

CAD

CARM1

CCDC115

CCDC22

CCT3

CD276

CD99

CDC42

CDK3

CDK5

CDK5RAP3

CELF2

CGI-59

CGN

CHD3

CHD5

CHMP4B

CHMP5

CLTB

CLUH

CNDP2

COASY

COG1

COG3

COMMD4

COMMD9

COPG2

COPS3

COPS7B

COQ7

COX5A

COX6A1

COX6A1P2

CPPED1

CPT1A

CSNK2B

CSNK2B-LY6G5B-1181

CSNK2B-LY6G5B--991

CSRP1

CSTF1

CTBP1

CTNNA1

CTNNA2

CTPS1

CTPS2

CUEDC1

CYB5R3

DARS

DBN1

DCTN2

DDAH1

DDB1

DDX17

DDX23

DDX24

DDX42

DDX5

DDX52

DDX5-ETV4 fusion

DHRS1

DIABLO

DISC1

DKFZp313D0632

DKFZp434M052

DKFZp434M0813

DKFZp434N1827

DKFZp434O1172

DKFZp564P2062

DKFZp666G172

DKFZp667O202

DKFZp686A01173

DKFZp686B20267

DKFZp686C0249

DKFZp686D0880

DKFZp686E1893

DKFZp686G1650

DKFZp686L02273

DKFZp686M19106

DKFZp762A1314

DKFZp781C0419

DKFZp781J074

DKFZp781K1356

DLST

DNAJA4

DNAJC8

DNM1L

DNPH1

DYNC1H1

DYNLT1

EDC4

EEF1D

EEF1E1

EFTUD1

EFTUD2

EHD1

EIF2A

EIF2AK2

EIF3F

EIF3G

EIF3M

EIF3S4

EIF4B

ELAVL1

ELMOD2

EMC1

EMC10

EMD

ENSA

EPCAM

EPHA2

EPHA4

EPHA6

EPHB1

EPHB2

EPHB2 variant protein

EPHB3

EPHB4

ESYT2

ETF1

ETFA

EVL

F11R

FADD

FAM203A

FAM203B

FAM83B

FANCI

FDXR

FLII

FLJ00053

FLJ00064

FLJ00283

FLJ00353

FLOT2

FLT1

FLT3

FLT4

FXN

FXYD3

GALE

GAPVD1

GBE1

GCLM

GFM1

GIGYF2

GLB1

GLOD4

GMFB

GNAI2

GNAL

GNAS

GNL3

GOLPH3

GOLPH3L

GPD2

GRPEL1

GSK3B

GSTZ1

GTF3C3

GYG1

HADHB

HDHD3

HDLBP

HEATR2

HECTD1

hEMMPRIN

HGS

HID1

HIGD2A

HINT1

HKE2

HMGCL

HMGCS1

HMOX2

HNRNPA3

HNRPA3

HSPA4L

HSPBP1

HSPH1

HSU53209

HTATIP2

IAH1

IER3IP1

ILVBL

IPO7

IRP1

JAK1

KDM3B

KDR

KHSRP

KIAA0196

KIAA1033

KIF3A

KIN27

KIT

KRT20

KRT222

L2HGDH

LAGE3

LANCL1

LEMD2

LGALS3BP

LIMK2

LIN7C

LOC84661

LONP1

LRRC41

LRRFIP1

LTA4H

LUC7L

MAP2K1

MAP2K2

MAP4

MAPK15

MCCC1

MCM3

MCM7

MDH1

METAP1

METAP2

METTL1

MGMT

MICU1

MIOS

MLL/CALM fusion

MLLT4

MOCS2

MOCS3

MPST

MRPL27

MRPL46

MRPL53

MSH2

MSRB2

MST4

MTOR

MYH9

MYO1D

NAA38

NACA2

NAP1L1

NAP1L4

NAPA

NAPRT1

NASP

NAT10

NCAPD2

NCAPG

NDUFA10

NDUFA5

NDUFS1

NIPSNAP1

NLK

NMNAT1

NNT

NOC4L

NOL9

NOP2

NOP9

NRBP1

NSDHL

NSF

NSUN2

NT5C2

NUMA1

NUP155

NUP214

NUP35

NUP37

NUP88

NUP93

OLA1

OPA1

ORMDL1

ORMDL2

OSBPL8

OTUB1

OXCT

OXCT1

P14

PAM16

PCNA

PDCL3

PDGFRA

PDGFRB

PFAS

PFDN1

PFDN6

PFKP

PGM2

PHKA1

PICALM

PIG43

PIG45

PITHD1

PITPNA

PITRM1

PLCB3

PLIN3

PMM2

PNP

POLD1

POLR2H

PPID

PPP2R1B

PPP2R5E

PPP5C

PPP6R3

PPT1

PRKACA

PRKAR2A

PRKCBP1

PRKCD

PRPSAP1

PRPSAP2

PSIP1

PSMB7

PSMD1

PSMD10

PSMD4

PSMD5

PSMD7

PTCD3

PTPMT1

PTPN11

PUS7

PYCR1

QARS

RAB25

RAB5A

RAB5B

RAE1

RALY

RANBP2

RB1

RBBP7

RBM12B

RBM19

RBM28

RBM45

RDH11

RDX

RFC3

RHPN2

RMDN1

RNF20

ROCK2

RPL17

RPL23

RPL31

RPRD2

RPS15A

RPS2

RPS4Y2

RRBP1

RRS1

SAC3D1

SAE1

SBDS

SCCPDH

SCFD1

SCYL1

SEC22B

SEC24C

SEC63

SEL1L

SEPHS2

SF3B1

SF3B14

SF3B2

SF3B3

SHROOM2

SHROOM3

SLC25A10

SLIRP

SLTM

SMARCC2

SMC2

SMC3

SMEK1

SMYD3

SNRPA

SNRPA1

SNRPE

SNRPF

SNX24

SPTBN1

SPTLC2

SQRDL

SRI

SRPRB

SRSF10

SSB

SSH3

SSR3

STK24

STX16

STX16-NPEPL1

STXBP2

SUCLA2

SUCLG2

SUGT1

SUMO2

SUMO3

SUMO4

SYNJ2BP

TACSTD2

TAGLN3

TBCE

TBRG4

TELO2

TES

TEX10

TFG

TFG/ALK fusion

THOC2

THOC7

TIMM50

TMEM209

TOLLIP

TOM1L2

TOR1A

TOR1AIP1

TOR3A

TPP1

TRA2A

TRAPPC6B

TRIM16

TRMT112

TRMT1L

TRMT5

TROVE2

TRRAP

TSNAX

TST

TSTA3

TTYH3

TUBG1

TUBG2

TWF1

TWF2

TXNRD1

TYSND1

UAP1

UBE2K

UBR4

UGP2

UQCRFS1

UQCRFS1P1

USMG5

USP15

USP9X

VAT1

VDAC3

VPS26A

VPS29

VTN

WASF2

WIBG

WTAP

XPO5

XPOT

YES1

YLPM1

ZFR

ZMPSTE24

ZMYND8

ZRANB2

ZYX

**Supplementary Table S3: List of proteins that were downregulated in MCF-7 following JA treatment (log_2_ ratio of treatment/control < -3)**

44M2.1

ABI1

ACACA

ACADVL

ACAP1

ACOT13

ACOT7

ACP1

ACTL6A

ACTR1A

ADSL

AIMP1

AKAP1

ALDH16A1

ALDH6A1

ANXA11

ANXA3

ANXA5

AP1G1

AP3M1

APOA1BP

ARF6

ARFGAP2

ARFIP1

ARHGAP1

ARL3

ARPC1A

ARPC1B

ARPC5

ASL

ASNS

ATAD2B

ATL2

ATL3

ATP2A1

ATP6V1G1

ATP6V1G2

ATP6V1G2-DDX39B

ATXN10

bA251O17.4

BCMO1

BID

BLVRB

BRK1

BRWD1

BUB3

BZW1

C16orf80

C19orf43

CA12

CALM1

CAND1

CAND2

CANX

CAPG

CAPN2

CARM1

CCAR2

CCS

CCT8

CDH1

CECR5

CETN2

CFDP1

CHMP2A

CKAP5

CLTA

CMPK

CMPK1

CMSS1

CNOT1

COA6

COMMD3

COMMD3-BMI1

COMMD5

COPG1

COPS2

COPS8

CORO1C

COX5B

COX7C

CPSF2

CRABP2

CROP

CSDE1

CSK

CTNND1

CYP1B1

DAZAP1

DAZAP1/MEF2D fusion

DCLK1

DCTN2

DDOST

DDX50

DEK

DES

DHX38

DIAPH1

DIP2B

DKC1

DKFZp564M2223

DKFZp586G1518

DKFZp666H126

DKFZp667H197

DKFZp686F2281

DKFZp686J0330

DKFZp686P17171

DKFZp761L1314

DKFZp761N09121

DKFZp779G2251

DKFZp779O2152

DKFZp781O1323

DLST

DNAJB1

DNAJC11

DNM2

DNMT1

DPP3

DPYSL2

DRG2

DTYMK

DYNLRB1

DYNLRB2

EIF1

EIF2A

EIF2B1

EIF2S2

EIF3D

EIF3H

EIF3K

EIF3L

EIF3M

EIF3S3

EIF4G3

ENY2

EPB41L4B

EPB41L5

EPS15L1

ERO1L

ERP29

ESRP2

EWSR1

EWSR1/ATF1 fusion

EWSR1/ZNF384 fusion

EXOC5

EXOSC3

FAM21A

FAM84B

FAM98A

FAM98B

FBP2

FIGNL1

FKBP1A

GAA

GABPA

GABPAP

GATA3

GCS1

GCSH

GEMIN5

GFRA1

GLB1L

GMPPB

GNB4

GNL1

GOSR2

GPS1

GSR

HAGH

HARS2

HBS1L

hCG_2023567

HEXA

HIBCH

HIP1R

HLTF

HNRNPLL

HNRPA1

HNRPLL

HSPA14

HSR1

HTATSF1

IARS

IDI1

IFP38

ILF3

IMPDH2

INPP4B

IPO8

IRS1

IRS-1

ITGA2

IVD

KARS

KDM1A

KIAA0101

KIF3B

KRAS

KTN1

LAMP2

LAMTOR5

LAS1L

LCMT1

LGALS3BP

LIMA1

LLGL2

LOC100996643

LOC727761

LUC7L

LUC7L3

M6PR

MADH5

MARCKSL1

MARS

MAT1A

MATR3

mcdrh

MCM4

MMSADHA

MOGS

MRPL21

MRPL43

MRPS18C

MRPS22

MRPS23

MRTO4

MTA2

MTHFD1L

MTHFD2

MVD

MYL6

MYO1B

MYOF

NAA25

NAA50

NAPA

NDFIP1

NDRG2

NDUFS7

NGDN

NIFK

NIPSNAP3A

NIT2

NPEPL1

NPEPPS

NPEPPSL1

NSFL1C

NUBP2

NUCKS

NUCKS1

NUDT16L1

NUMB

NUP188

NUP214

NUP88

NUP93

OAT

OGT

OK/SW-cl.65

ORF1

P4HB

PABPC1

PAK1

PALLD

PAWR

PC

PCK1

PDCD6

PDHB

PDLIM5

PDS5A

PEA15

PEX11B

PFDN5

PFN2

PGM3

PGRMC1

PHIP

PIGT

PIGU

PLAA

PLCG1

PLCG1 variant protein

PLS3

PNPO

POLR1A

POLR2A

POLR2I

POP1

POR

PPIF

PPM1A

PPM1B

PPP1CC

PPP2R4

PRDX4

PRKAR2B

PRPF4

PRPH

PRPS1L1

PRPSAP2

PSMA3

PSMD6

PSMD8

PSME3

PSMG1

PSPH

PTGR2

PTMS

PTTG1IP

PWP2

PWWP2A

PYGB

QTRTD1

RAB13

RAB27B

RAB2A

RAB2B

RABGAP1

RAD21

RAD23A

RALB

RANGAP1

RAP2C

RBM12

RBM15

RBM4

RBM4B

RDX

RFC4

RPL15

RPL18A

RPL36

RPL39

RPL39P5

RPL5

RPN1

RSL1D1

RSU1

RTCB

S100P

SARNP

SCAMP3

SEC23IP

SEPT9

SERBP1

SERPINA3

SERPINB1

SF1

SF3A1

SF3B4

SFPQ

SFXN3

SH3GLB2

SKIV2L2

SKP1

SLIRP

SMAD1

SMAD2

SMAD3

SMAD5

SMAD9

SMARCA1

SMARCA5

SMCHD1

SMU1

SNAP29

SNRNP40

SNRPD2

SNW1

SNX4

SPTAN1

SRM

SRP72

SRP9

SSRP1

STAU1

SYPL

SYPL1

TAF15

TARDBP

TBC1D3

TBCA

TCEB2

TCERG1

TCP1

TERF2IP

TFF1

THUMPD1

TIMM8A

TJP3

TMEM214

tmp_locus_29

TMSB10

TNPO1

TOM1

TOP1

TPD52

TRAF2

TRAPPC3

TRIM24

TSN

TSR1

TUBB3

TXNDC

U2AF1L4

UBA2

UBA3

UBE2Z

UNQ1844

UQCRQ

USP14

USP8

UTP18

VIM

VPS16

VPS45

VPS4A

VPS4B

WBP11

WDR4

WDR57

WDR77

WDR9

YBX2

YY1

ZDHHC7

ZNF607

ZNF706

**Supplementary Table S4: List of enriched shRNAs**

A1CF

ABCB9

ABCC1

ABP1

ACAD9

ACIN1

ACMSD

ACY1

ADC

ADCK1

ADH4

ADH6

AGMAT

AK4

AKAP11

ALDH1A3

ALDH3A1

ALDH3A2

AMD1

ANKRD40

ANO6

AP1S2

AQR

ARHGEF25

ATF7IP2

BLMH

BRDT

C11orf46

C19orf43

C1orf55

C20orf158

C9orf11

CALR3

CAMKK2

CAP2

CCDC68

CCR2

CDC42BPA

CDC7

CDK15

CDKL4

CDS2

CELF2

CELF4

CENPA

CHDH

CLK1

CMPK1

COL4A3BP

COPS2

CPM

CPSF2

CPSF3

CSNK1G1

CSNK2A1

CTNNB1

CWC22

CYTL1

DDR2

DDX17

DDX6

DERA

DISC1

DLST

DNAJC17

DSPP

DUOX1

DUSP19

DYRK1B

EEF2K

EFHA1

EFTUD2

EGR2

EIF2AK3

EIF2S2

EIF3I

EIF4A3

EMP1

EPHA6

ERI2

EXT1

FADD

FAM122B

FAM26D

FAM32A

FAM81A

FASTK

FER

FGF5

FGFR2

FIP1L1

FLJ16124

FYCO1

GABRA6

GAD1

GAD2

GAPDH

GLS

GLS2

GLUD1

GLUD2

GPATCH1

GPD2

GPR113

GRSF1

GSG2

GSK3B

GSPT1

GSTA3

GUCY1B2

HADHB

HAT1

HMGCS1

HNRNPA3

HNRNPH3

HNRNPUL1

HSD17B7

HSPA1L

HUNK

IFT46

IGF2BP3

IL1F8

IL31

INSRR

IRAK4

IRS1

ITCH

ITGB3

JAK1

JMJD7-PLA2G4B

KAT2A

KDM6A

KHDRBS1

KIAA1429

KIAA1958

KIF16B

KLK5

KLKB1

KRT23

LACTB

LDHAL6B

LOC391513

LOC401313

LOC402677

LRP6

LRRK2

LSM11

LSM5

LSM6

MAOA

MAP3K1

MAP3K7

MAP4K2

MAP4K5

MAPK10

MAPKAPK2

MARK1

MARK3

MASTL

MBIP

MCCC1

MCCC2

MCTP1

MCTP2

ME1

ME2

MELK

MET

MFSD11

MICALL1

MKI67IP

MMP27

MSH5

MST1R

MST4

MTHFD2L

MXRA8

MYO3A

NAA15

NAA38

NCOR1

NDST2

NDUFA4

NDUFAF1

NDUFB6

NDUFB9

NEK1

NEK10

NINL

NNT

NPR1

NR3C2

NT5C2

NTF3

NTRK2

NTRK3

ODC1

OR10X1

P4HA2

P4HA3

PABPC1

PABPC4

PAK1

PAK3

PAN3

PAPOLG

PC

PCBP2

PCBP3

PCCA

PCCB

PDE5A

PDHA2

PDILT

PGD

PGK1

PGR

PHF5A

PIK3C2A

PIK3C2B

PIM2

PLCB2

PLCG2

PLCL1

PLCL2

PLCXD2

PLCZ1

PLOD3

PLXNC1

PPAT

PPM1J

PRCC

PRKAA2

PRKACB

PRKAR1B

PRKCG

PRLR

PRMT3

PRODH2

PROM1

PRPF3

PRPF4

PRPF6

PSAP

PSIP1

PTGR2

PTK6

RAB9B

RAC1

RAF1

RALA

RALYL

RASA2

RASSF3

RASSF6

RBL1

RBM10

RBM12

RBM15

RBM8A

RCOR3

RDH11

RDH5

REL

REXO1

RIC8B

RNF180

ROCK1

RPE

RPL12

RPL13

RPL18A

RPL21

RPL22

RPL23A

RPL39

RPL4

RPL5

RPLP0

RPS10

RPS11

RPS17

RPS18

RPS25

RPS27

RPS3A

RPS4X

RPS4Y1

RPS4Y2

RPS6KA4

RPS6KC1

RPS7

RPS8

RSL24D1

RWDD3

RYK

SAFB2

SAT1

SCYL3

SEC14L1

SF3B1

SF3B3

SF3B5

SGK2

SGK3

SIK2

SLC16A6

SLC1A5

SLC5A9

SMPDL3B

SMS

SMU1

SNIP1

SNRNP40

SNRPA

SNRPD2

SNRPD3

SNRPE

SNRPF

SNRPG

SNW1

SRRT

STK31

STK33

STRADB

SUGP1

SUZ12

SYT16

SYTL5

TAAR8

TAF1

TALDO1

TAOK1

TARS2

TAS1R2

TAS2R19

TBX5

TCEB1

TEK

TENC1

TET1

THOC5

TIA1

TJP2

TKT

TKTL1

TLK1

TMEM20

TNNI3K

TP53BP1

TRAF6

TSPAN1

TTBK1

TUBB

TYK2

UGT8

UHMK1

UNC13B

UROD

USP11

USP17L6P

USP38

USP47

USP50

USP54

USP6

VANGL1

VPS29

WBP11

WDR33

WNK2

WNK3

WSB2

XAB2

ZC3HC1

ZFC3H1

ZIM2

ZNF10

ZNF331

ZNF568

ZNF658

**Supplementary Table S5: List of depleted shRNAs**

ABCD3

AGXT2L1

ARHGAP15

ATG4A

ATG4C

ATP9B

ATRNL1

ATXN7

BTG3

BTG4

C10orf12

C16orf5

C1orf124

C3orf19

C4orf43

C6orf10

CCNT2

CD40

CDH20

CHD9

CIR1

CLCA4

CLPTM1

CREB3L2

CXADR

CYP51A1

DECR1

DOPEY2

DUSP27

E2F6

ENTPD2

EPB41L4B

FAM169B

FEZ2

GALT

GDF5

GFRA1

GK

GMNN

GPR174

GPS2

GRIK3

H1FOO

HDHD2

HOXA11

HUS1

IL1B

IL1F5

IMPDH2

ITPR2

KRTAP10-6

LAPTM5

LCT

LEP

LGSN

LHPP

LIG1

LOC401198

LRP10

M6PR

MAN2B2

METAP1

MEX3B

MOCS3

MSN

NAMPT

NCRNA00288

NKX3-1

NOS3

NPHP3

NPSR1

NRL

OR51D1

P2RX1

PDE12

PDE1C

PES1

PIGU

PLEKHA5

PPFIA2

RAB36

RAB39B

RASGRP3

RHOBTB2

RNF150

ROBO1

RUFY3

SAMD9

SDHDP7

SERPIND1

SH2B3

SLC16A14

SLC17A8

SLC2A2

SLC4A5

SMARCA1

SMOC1

SOS2

SPINK14

SREBF2

SRPX

SSX7

SSX9

STX7

TACR1

TAS2R42

TFG

TMEM120A

TNFRSF11B

TOM1L2

TRIT1

UBE2E4P

UBLCP1

VPS36

WDR45L

WFDC3

WIF1

ZFP42

ZNF441

ZNF510

ZNF620

**Supplementary Table S6: List of targets identified from the combined proteomic-RNAi screen**

| **Genes/Pathways** | **Gene symbol** |
| --- | --- |
|  |  |
| **Spliceosome** |  |
| splicing factor 3b subunit 1 | SF3B1 |
| heterogeneous nuclear ribonucleoprotein A3 | HNRNPA3 |
| elongation factor Tu GTP binding domain containing 2 | EFTUD2 |
| small nuclear ribonucleoprotein polypeptide A | SNRPA |
| small nuclear ribonucleoprotein polypeptide F | SNRPF |
| N(alpha)-acetyltransferase 38, NatC auxiliary subunit | NAA38 |
| small nuclear ribonucleoprotein polypeptide E | SNRPE |
| splicing factor 3b subunit 3 | SF3B3 |
|  |  |
| **Valine, leucine and isoleucine degradation** |  |
| methylcrotonoyl-CoA carboxylase | MCCC1 |
| 3-hydroxy-3-methylglutaryl-CoA synthase 1 | HMGCS1 |
| hydroxyacyl-CoA dehydrogenase, beta subunit | HADHB |
|  |  |
| **Nicotinate and nicotinamide metabolism** |  |
| nicotinamide nucleotide transhydrogenase | NNT |
| 5'-nucleotidase, cytosolic II | NT5C2 |
|  |  |
| **Pathways in cancer** |  |
| glycogen synthase kinase 3 beta | GSK3B |
| Janus kinase 1 | JAK1 |
| Fas associated via death domain | FADD |
|  |  |
| **Others** |  |
| serine/threonine protein kinase 26 | STK26 |
| DEAD-box helicase 17 | DDX17 |
| PC4 and SFRS1 interacting protein 1 | PSIP1 |
| acyl-CoA dehydrogenase family member 9 | ACAD9 |
| disrupted in schizophrenia 1 | DISC1 |
| CUGBP, Elav-like family member 2 | CELF2 |
| EPH receptor A6 | EPHA6 |
| retromer complex component | VPS29 |
| glycerol-3-phosphate dehydrogenase 2 | GPD2 |
| dihydrolipoamide S-succinyltransferase | DLST |
| retinol dehydrogenase 11 (all-trans/9-cis/11-cis) | RDH11 |
| ribosomal protein S4, Y-linked 2 | RPS4Y2 |

**Supplementary Table S7: Forward and reverse primer sequences for quantitative RT-PCR.**

| **Target Genes** | **Sequence (5’-3’)** | |
| --- | --- | --- |
|  | **Primer (Forward)** | **Primer (Reverse)** |
|  |  |  |
| **mRNA qPCR (span exon/exon junctions)** | | |
| **SF3B1** | TTGGGAGCGCCAGTTCCGT | GATCAGGGGTTTTCCCTCCATCT |
| **SF3B2** | GACCACCCCCATCGTATCCC | AGGTGTCTCACTTCCGTCCATC |
| **SF3B3** | ATGCCTCTGGAAGAAGGAGACACA | TGCCATCTGGATAGACCTGCACC |
| **SF3B4** | GGGCTTCCTCCACCAGGCAT | GCGTGGGGATGTCCTAAGCC |
| **SF3B14** | GTCTCGCTCAGGCTCGGTTT | AAGTCGAATGTTCGCCCTCTTGG |
| **RIOK3** | TGCATATGGAGGGAGCATGG | AGCTCTCTGCATTCTTGCGA |
| **BRD2** | TAGCCAGCCCCAGAGGATTA | GTGACACAGAAGAGACGGCA |
| **DNAJB1** | CTTCGGTGGCAGAAATCCCT | CAGCCACACAGAGCCTCC |
| **CDKN1B** | CAGCTTGCCCGAGTTCTACT | AGAAGAATCGTCGGTTGCAGG |
| **GAPDH** | GTCTCCTCTGACTTCAACAGCG | ACCACCCTGTTGCTGTAGCCAA |
|  |  |  |
| **End-point PCR (span 2 exons)** | | |
| **RIOK3** | TCCAGTGACCTTATGCTGGC | TGGTCTGTAGGGATCATCACG |
| **BRD2** | GACCACGGTGGCCAAAATTC | TAAATGTAACAGTTGGTGAACATGG |
| **DNAJB1** | TAAACCCCGACGGAAAGAGC | AGTCCTTGGGGAGCTCAGAT |
| **CDKN1B** | GCTAACTCTGAGGACACGCA | TTTGACGTCTTCTGAGGCCA |
|  | | |
| **Unspliced mRNA PCR (span intronic region)** | | |
| **RIOK3 intron 3** | TCAATGGAGATAGCAAAGGTATTATAAC | AGATTTACTTAGGAGCACATTATGAGTG |
| **BRD2 intron 4** | AGGTAATGTCACAGGATGGGAAGT | CCCTGCTGCCTTTCTCTAACC |
| **DNAJB1 intron 2** | GGCCTGATGGGTCTTATCTATGG | TTAGATGGAAGCTGGCTCAAGAG |
| **CDKN1B intron 1** | GTAATGACCCTTTCCCAACCATAG | CTAAGGTTAACACCCTCCAGCAG |

**Supplementary Table S8: shRNA target sequences for SF3B1 and SF3B3**.

|  | **Target sequence** |
| --- | --- |
| **SF3B1si-1** | 5'-TGCTTTGATTTGGTGATGTAA-3' |
| **SF3B1si-2** | 5'-CAACTCCTTATGGTATCGAAT-3' |
| **SF3B3si-1** | 5’-GTTGGAGTAGATGTCGGATTT-3’ |
| **SF3B3si-2** | 5’-TGAGAGTAACAACCTTATTAT-3’ |

**Full-length gels**


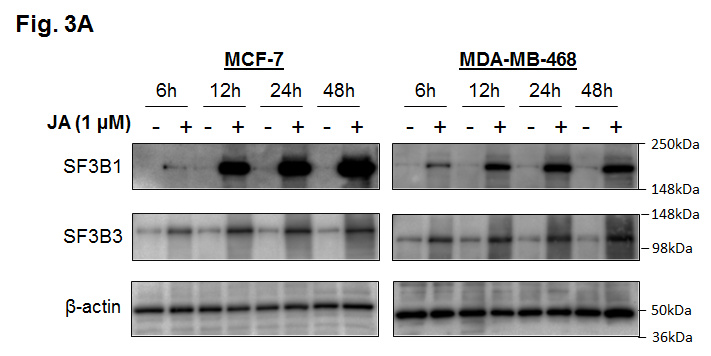


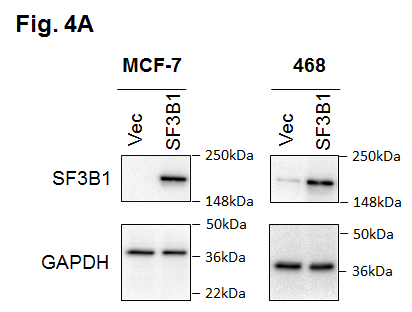


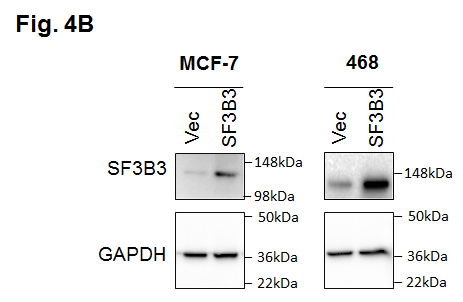


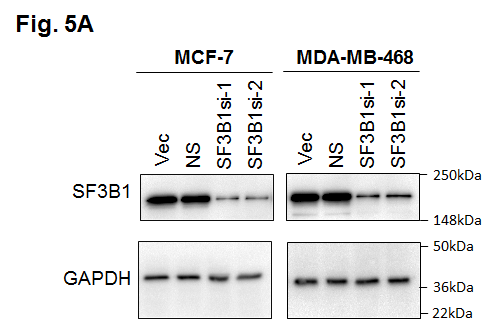


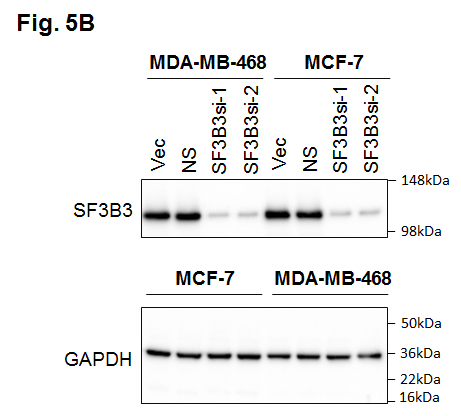


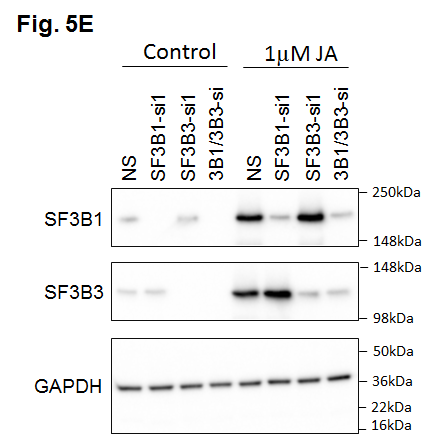


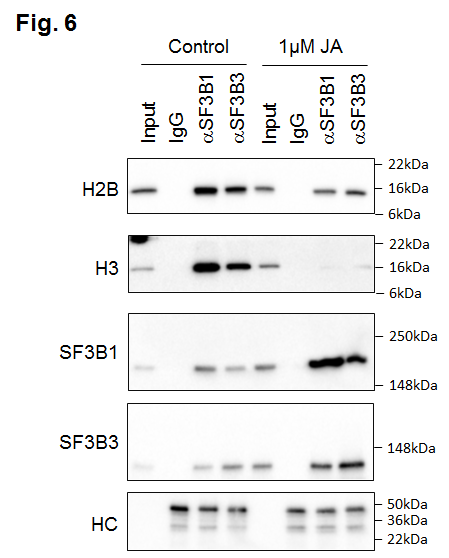

Supplement: Supplementary Information [file srep42504-s1.doc]
